# A Novel Approach of SWATH-Based Metabolomics Analysis Using the Human Metabolome Database Spectral Library

**DOI:** 10.3390/ijms231810908

**Published:** 2022-09-18

**Authors:** Hassan Shikshaky, Eman Abdelnaby Ahmed, Ali Mostafa Anwar, Aya Osama, Shahd Ezzeldin, Antony Nasr, Sebaey Mahgoub, Sameh Magdeldin

**Affiliations:** 1 Proteomics and Metabolomics Research Program, Basic Research Department, Children’s Cancer Hospital Egypt 57357, Cairo 11441, Egypt; 2 Department of Pharmacology, Faculty of Veterinary Medicine, Suez Canal University, Ismailia 41522, Egypt; 3 Department of Physiology, Faculty of Veterinary Medicine, Suez Canal University, Ismailia 41522, Egypt

**Keywords:** data-independent acquisition, high-resolution LC-MS, metabolomics, MS/MS library, SWATH

## Abstract

Metabolomics is a potential approach to paving new avenues for clinical diagnosis, molecular medicine, and therapeutic drug monitoring and development. The conventional metabolomics analysis pipeline depends on the data-independent acquisition (DIA) technique. Although powerful, it still suffers from stochastic, non-reproducible ion selection across samples. Despite the presence of different metabolomics workbenches, metabolite identification remains a tedious and time-consuming task. Consequently, sequential windowed acquisition of all theoretical MS (SWATH) acquisition has attracted much attention to overcome this limitation. This article aims to develop a novel SWATH platform for data analysis with a generation of an accurate mass spectral library for metabolite identification using SWATH acquisition. The workflow was validated using inclusion/exclusion compound lists. The false-positive identification was 3.4% from the non-endogenous drugs with 96.6% specificity. The workflow has proven to overcome background noise despite the complexity of the SWATH sample. From the Human Metabolome Database (HMDB), 1282 compounds were tested in various biological samples to demonstrate the feasibility of the workflow. The current study identified 377 compounds in positive and 303 in negative modes with 392 unique non-redundant metabolites. Finally, a free software tool, SASA, was developed to analyze SWATH-acquired samples using the proposed pipeline.

## 1. Introduction

Progress and development in mass spectrometric technology have driven researchers into a new era of omic research—metabolomics. The whole profiling of small molecules (<1500 Da) present within an organism or cell, metabolome, moved us further toward depicting the immediate metabolic image of any living cell [1]. Consequently, it filled up the gap between genotyping and phenotyping. Therefore, the recruitment of metabolomics in clinical diagnosis has become more prominent in the last few years since any disease condition incorporates alteration in the expression of body metabolites [2]. In addition, it has shown great promise in a better understanding of molecular medicine, therapeutic drug monitoring, and drug development [3,4]. The reliable identification of metabolites in different biological samples substantially translates the analytical output into meaningful biological information [5]. This identification could only be accomplished using a high-quality spectral database annotated for metabolites [6].

Despite the existence of different databases, metabolite identification remains the bottleneck in metabolomics analysis owing to several facts: (1) the ample search space of analytes with broad dynamic range and structural resemblance resulting in mismatching, (2) the need for authentic standards for database construction, which are expensive and available for relatively few metabolites, (3) high fragmentation pattern variability resulted from different collision-induced dissociation (CID) observed in various web-based libraries with MS2 spectral databases such as HMDB, Metlin, MassBank, and LipidMaps. All these obstacles hinder accurate fragment matching and increase the false discovery rate [7,8].

The conventional metabolomics analysis pipeline depends mainly on the information-dependent acquisition (IDA) approach. Notwithstanding a powerful technique, it still suffers from several critical limitations. In particular, a stochastic non-reproducible selection of predefined highly abundant ions at the expense of low abundant ions [9]. Not all precursors become fragmented in a single experiment, which hinders the reproducibility and results in a less sensitive detection of molecules in a complex mixture [10].

The surrogate use of the data-independent acquisition (DIA) pipeline, such as the sequential windowed acquisition of all theoretical MS. (SWATH), has attracted much attention to overcome IDA drawbacks by leveraging all MS2 [11,12]. Previously, SWATH-based label-free proteomics quantification achieved a higher dynamic range and reproducibility of identification and improved sensitivity and accuracy of quantification than the IDA [13]. The burgeoning use of this approach relies upon two main features: the low chance of interference since the odds of two different molecules with the same MS1 and MS2 signature at high resolution is minimal, and acquiring all precursors enables identification and quantification without prior knowledge of the nature of the sample [10]. Therefore, the SWATH method has extensive coverage with reproducibility during sample analysis in addition to the complete MS2 spectra collection [14].

Notwithstanding, SWATH suffers from overcrowded spectra that require a robust curation to match fragment ions with their precursor [15]. During SWATH acquisition, fragment ions are acquired simultaneously, and the link between the precursor ion and its fragments is lost, so the fragments of co-eluting molecules must be separated to achieve a high alignment level [6,15]. Therefore, a highly curated SWATH library has become mandatory to mitigate this limitation [6]. Moreover, there is limited available open-access software for efficient use of SWATH datasets for untargeted metabolomic analysis. The metabolomics analysis includes feature generation, normalization, alignment, quantification, and transition list generation. Single software cannot perform all the pipeline steps because each step needs a different algorithm [13,15], so filling any node in this cycle is still needed. Various tools were developed to facilitate SWATH sample analysis. For instance, “MetDIA” is a metabolomic R language package that relies on the targeted extraction of small molecules from multiplexed MS2 spectra generated by DIA but is limited to only targeted analysis [14].

On the other hand, MS-DIAL was developed for untargeted metabolomics [16]. The platform depends on MS1 and MS2 spectral library identification [16]. However, the accuracy of MS-DIAL results was 20 to 75%, depending on the sample type [14]. “SWATHtoMRM” is an “R” package that is used for targeted analysis and uses an in-house library [17]. Limitations of this package encompass narrow SWATH window searches and require high computational power.

The major challenge in SWATH integration is the lack of software tools with efficient and reliable compound identification. Here, we developed a novel SWATH platform for non-targeted data analysis with an accurate mass spectral library for metabolite identification using SWATH acquisition. Data analysis is based on scoring precursor ions with their possible transition signals. This strategy is operated via an unbiased SWATH-based approach, in cyclic recording, over the acquisition time range of sequential survey scans and precursors transition ion spectra in predetermined isolation windows. The validated platform utilizes a mass spectral library of 1282 high-resolution small molecules parsed from the HMDB version 4.0 for both positive and negative modes. The fidelity of the workflow was tested using predesigned inclusion/exclusion and various biological samples: plasma, urine, and HL60 cell line. Finally, we designed a software tool named the SWATH-Auto System Analyzer Tool (SASA) to simplify our pipeline algorithm semi-automatedly. SASA is freely available on SourceForge (https://sourceforge.net/projects/sasatool/) accessed on 1 September 2022 with complete documentation and tutorials (https://www.57357.org/app/uploads/2019/12/SASA-documentation-1.pdf accessed on 1 September 2022). The SASA code is open access and available on (https://github.com/Proteomicslab57357/SASA) accessed on 1 September 2022.

## 2. Results and Discussion

### 2.1. Determining the Filtration Criteria

**Figure 1:**
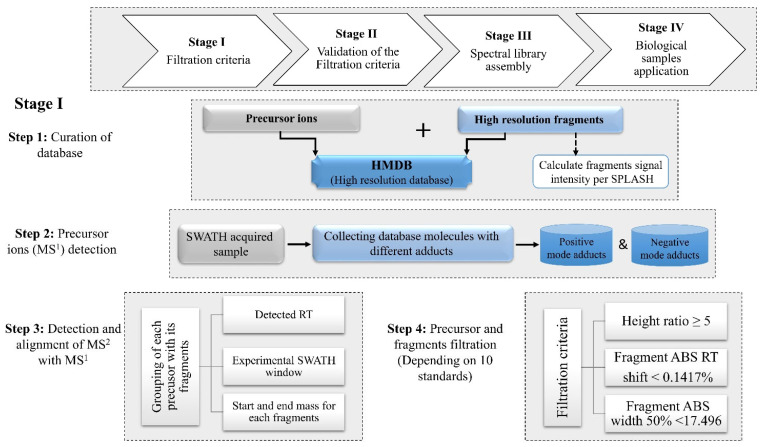
Experimental design and schematic illustration of the SWATH-Auto System Analyzer (SASA) workflow for small molecule extraction of SWATH-acquired samples. The pipeline of the filtration criteria (**Stage I**) includes four steps: (**Step 1**) curation of database (MS1, MS2, and the relative intensity); (**Step 2**) precursor ions (MS1) detection; (**Step 3**) detection and alignment of MS2 with MS1; (**Step 4**) precursor and fragments filtration.

The current study aims to develop a simple approach to discovering potential metabolic vulnerabilities. It utilizes the preferential aligning of predefined database fragments with robust screening using untargeted SWATH acquisition (Figure 1). This approach gathers both selectivity and sensitivity of identification together with the breadth of the dynamic range of SWATH acquisition [7].

**Figure 2:**
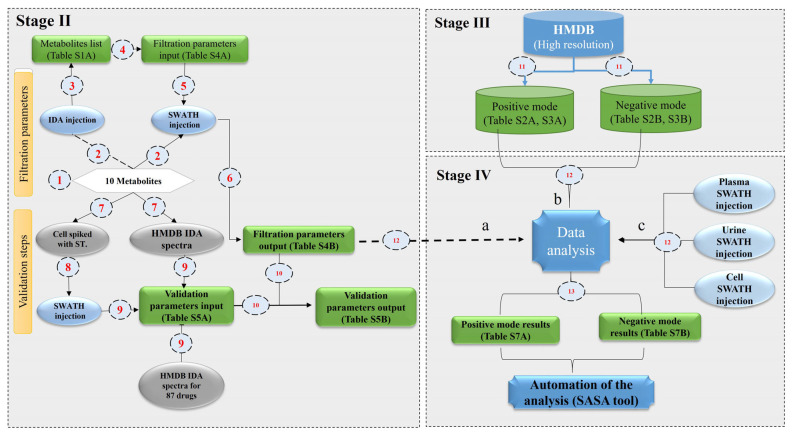
Validation, spectral library generation, and data analysis pipeline. **Stage II** clarifies the filtration and validation steps using the spiked-standard mixture in both IDA and SWATH analysis. **Stage III** explains HMDB version 4.0 spectral library generation. **Stage IV** demonstrates the strategy of the biological data analysis workflow. Green shaded boxes are described in the supplementary spreadsheet. Numbers 1–13 clarify the schematic workflow direction through the different stages. The letters a, b, and c explain the sequential data analysis.

Individual IDA injection for ten standards was used to compile MS1 and MS2 profiles together with retention time and relative intensities for setting the filtration criteria “Supplementary Table S4A; filtration parameters input” (Figure 2, stage II). These extracted criteria were applied for filtrating the SWATH-acquired standards. The gist of our novel method relies on identifying each analyte based on the mass accuracy of the precursor ion and matching its fingerprint fragments with the curated database from HMDB. This approach provides additional information about the analyte structure that could be used for library searching and/or de novo identification.

Each precursor ion is aligned with its product ions, as illustrated in Figure 1, and steps 2 and 3 are used to generate the “filtration parameters output” (Supplementary Table S4B and Figure 2, Stage II). The filtration parameters include height ratio in samples to blank ≥5, ABS retention time (RT) shift <0.1417%, and ABS FWHM for each fragment from its precursor ion <17.4965. These filtration data were used later for subsequent validation steps.

**Figure 3:**
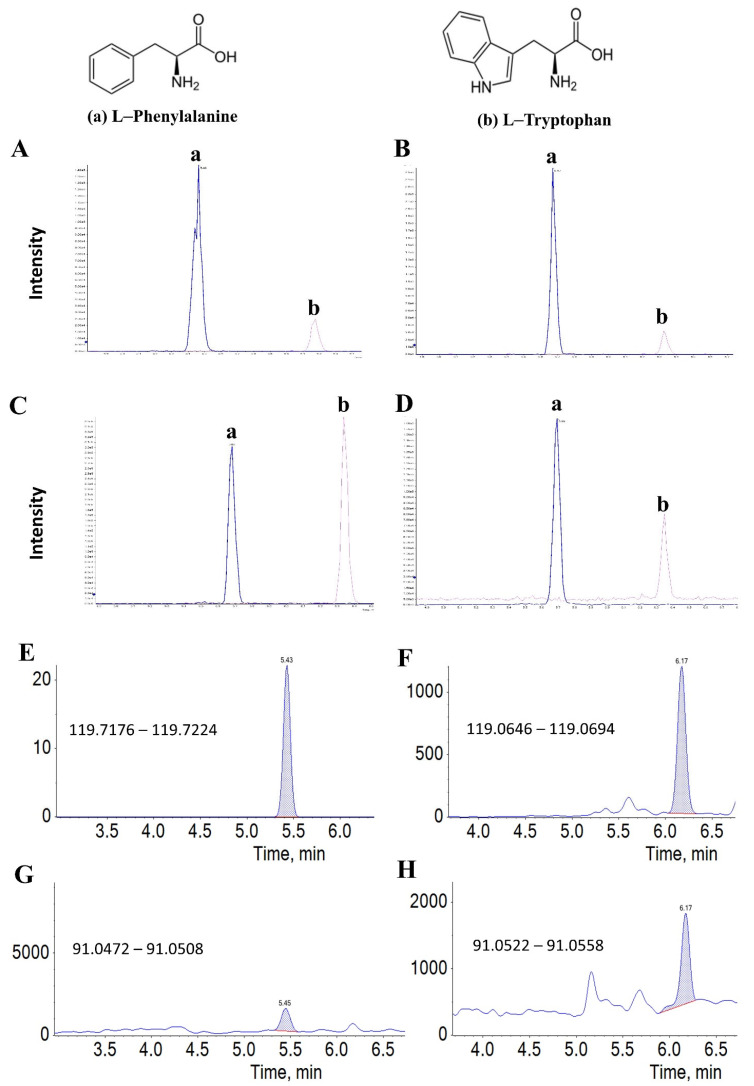
Accuracy of predefined filtration criteria. Two aromatic amino acids, including phenylalanine and tryptophan, spiked in (**A**) HL-60 cell extraction (**B**), urine (**C**), and plasma (**D**). Both molecules share a basic aromatic ring structure. The primary identification hitting target depends on the side chain fragmentation (**E**,**G**) for l-Phenylalanine and (**F**,**H**) for l-Tryptophan.

The selected authentic standards (Supplementary Table S1A) represent small molecules with different retention times and polarities usually reported in urine, plasma, and cells. To show the credence of accurate identification, the standard includes two aromatic amino acids (phenylalanine and tryptophan) that share a basic ring structure. In the HMDB database, phenylalanine shares more than 55% of its transitions with tryptophan (Supplementary Table S1B and Figure 3). The distinguishing between both molecules is challenging because it relies mainly on side chain recognition with slight differences in elution time. The current generated pipeline differentiated between these compounds even under the matrix effect of all tested biological samples, as clarified in Figure 3. While previous literature reported that some metabolites were erroneously identified when matched with precursor ion isolation due to the background noise or similar structure compound mismatching [18], the confirmatory MS2 fingerprinting at this moment minimizes the mismatching possibilities. Therefore, it is not the case anymore in our approach.

### 2.2. Validation of the Filtration Criteria

We tested the validity of the proposed pipeline together with the matrix effect of biological samples. Therefore, ten authentic standards (inclusion list) plus 87 non-biological synthesized pharmaceutical molecules (exclusion list) were used (Supplementary Table S6). Fragmentation ions of these 97 compounds were parsed from HMDB and listed as “Validation parameters input” (Supplementary Table S5A and Figure 2, Stage II). The experimental windows for the total 97 compounds were calculated depending on the SWATH acquisition window (50 Da), with the extraction window mass shift adjusted at 0.05 Da. Four early defined filtration steps were applied to the injected samples (Figure 2, Stage IV) to generate the “Validation parameters output” (Supplementary Table S5B and Figure 2, Stage II). Finally, ions with height ratio in samples to blank ≥ 5, ABS retention time (RT) shift < 0.1417%, and ABS FWHM < 17.4965 for each fragment from its precursor ion were retained. It is worth mentioning that stringent filtration criteria were applied to eliminate the likelihood of false-positive matches and attain maximum accuracy. Coherently, the standard MS2 transitions were well-matched with the generated consensus spectrum library, which attests to the validity of the workflow. Our results, “Validation parameters output”, showed that false-positive hits were 3.4% with 100% sensitivity and 96.55% specificity (Table S5B and Figure 2) in the case of a curated experimental database. The false-positive hits ranged from 20 to 25% when the pipeline ran on the predicted HMDB. This percentage depends on the nature of the sample.

**Figure 4:**
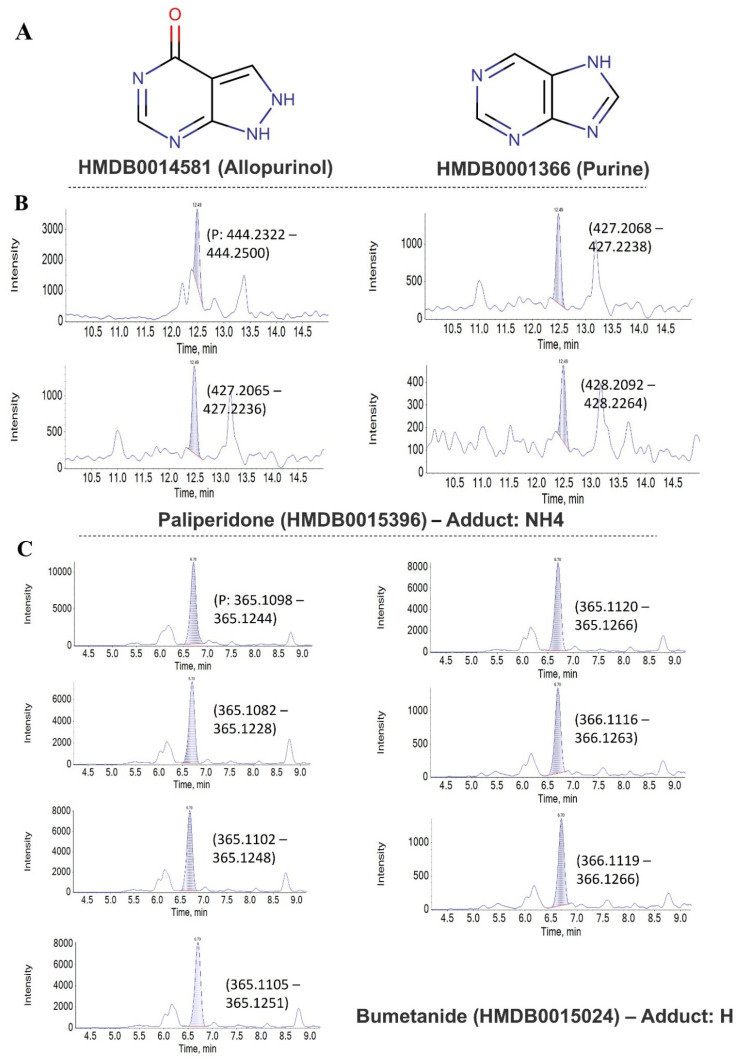
False-positive identified compound: (**A**) clarifies structure similarity, (**B**) the parent with the ammonium adduct without further fragmentations, and (**C**) the protonated form of the compound.

Curiously, the three false-positively identified compounds were allopurinol, bumetanide, and paliperidone. Allopurinol is an analog of the naturally occurring purine, the isomer of hypoxanthine (Figure 4A). Notably, these drugs resemble naturally endogenous compounds and almost share the same MS2 transitions. On the other hand, we noticed that false-positive identifications were due to poor fragmentation of the compounds due to their chemical nature (Figure 4B,C) as the compounds expressed in the neutral form or the formation of different adducts, either ammonium or protonated form without any further fragmentations. Even the compounds have other fragments, but they all represent the same products with narrow mass shifts.

Some open-source algorithms were developed, such as MS-DIAL, MetDIA, and SWATHtoMRM, to analyze SWATH-based approaches. MS-DIAL filtration criteria rely mainly on retention time and the mass accuracy of MS1 and MS2 [16]. Recently, Shah et al., 2022, reported that none of the MS2 spectra for the ten added standards were deconvoluted correctly with their reported fragment ions and retention time. In addition, serum metabolites identified in DDA (IDA) were also not correctly deconvoluted in DIA (SWATH) mode using MS-DIAL [10]. Moreover, another issue facing open-source tools can be the conversion of data from the vendors to an open format, which makes the result problematic as metadata is not included in the final files, and data quality can suffer during conversion [5].

Further, the conversion process converts the already large SWATH raw files to much larger files [5], and this problem is very apparent in the “SWATHtoMRM.” Another study reported that the accuracy of the MS-Dial result was 20 to 75% less than MetDIA [14]. The latter depends on the targeted extraction of small molecules from multiplexed MS2 spectra generated by data-independent acquisition. The software was evaluated using a set of 30 metabolites from the human metabolome database, and all metabolites were identified with a 1% false-positive rate. However, the latter validity was limited to targeted analysis only, where the spectral library consisted of 786 analytes.

Moreover, Zha et al. developed an “R” package termed “SWATHtoMRM” used for targeted analysis that depends mainly on an in-house library [17]. The method was applied to build a targeted metabolomics method that identified 1303 metabolites. In addition, it needs high computational capability during processing.

In this approach, data analysis is based on scoring the alignment probability of a given precursor with its possible transition signals. One advantage now is that any desired database could be generated through the software with different SWATH window tolerance. Overcoming the drawbacks mentioned above, the current pipeline not only can analyze a large number of compounds using the SWATH method but data could also be reanalyzed for in-depth information whenever the database is updated or expanded [19].

### 2.3. Fragmentation Efficiency of SWATH and IDA Compared to HMDB

**Figure 5:**
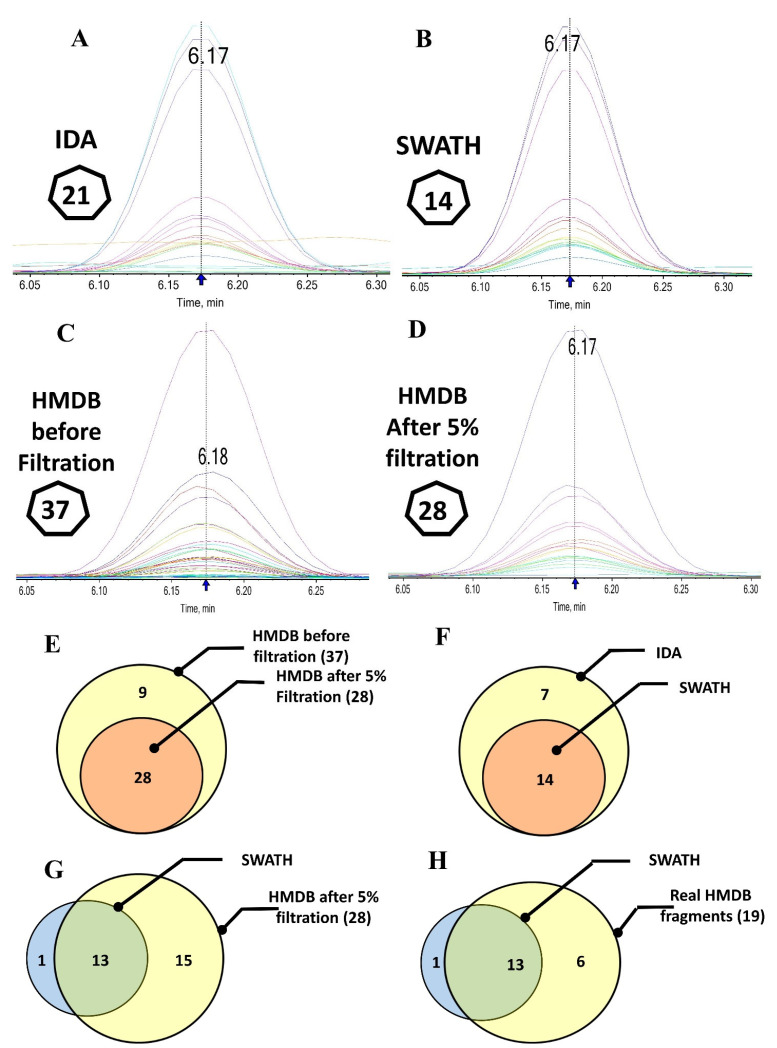
Comparison and overlapping between tryptophan fragmentation pattern spectra of SWATH with IDA and HMDB (each color spectra represent different fragments). It shows IDA (**A**) and SWATH fragments (**B**), while (**C**,**D**) clarify fragments present in the HMDB before and after filtration. (**E**–**H**) represents their overlapping.

Fragments were aligned to compare the efficiency of the fragmentation pattern generated from SWATH-acquired samples with IDA fragmentation and the HMDB workbench (Supplementary Table S6, Figure 5). Tryptophan gave 21 and 14 fragments when the sample was acquired in IDA (Figure 5A) and SWATH modes (Figure 5B,F), respectively. Manual inspection of these fragments disclosed that the seven missed fragments were background-related noise.

Tryptophan transitions in HMDB showed 37 fragment ions (Figure 5C). After filtration of these transitions with more than 5% relative intensity, only 28 (75.7%) transitions remained (Figure 5D,E). Other 9 (24.3%) were recognized as background noise. Simultaneously, 9 transitions out of the 28 filtered peaks were repeated with a slight mass shift. This is because HMDB pools data from different instruments and settings. Accordingly, the SWATH workflow identified all the base peak transitions tagged as “D” and 68.5% of the real (19; Figure 5G,H) transitions collectively (see Supplementary Table S6).

This result reveals our approach’s fidelity to picking up the real spectra and subtracting background noise despite the complexity of the SWATH sample (Supplementary Table S7A,B). In the next step, the remaining identified transitions with high confidence were used for subsequent confirmatory steps. In parallel, manual validation at this stage illustrated a perfect alignment of fragment ions with their precursor.

### 2.4. Library Generation

**Figure 6:**
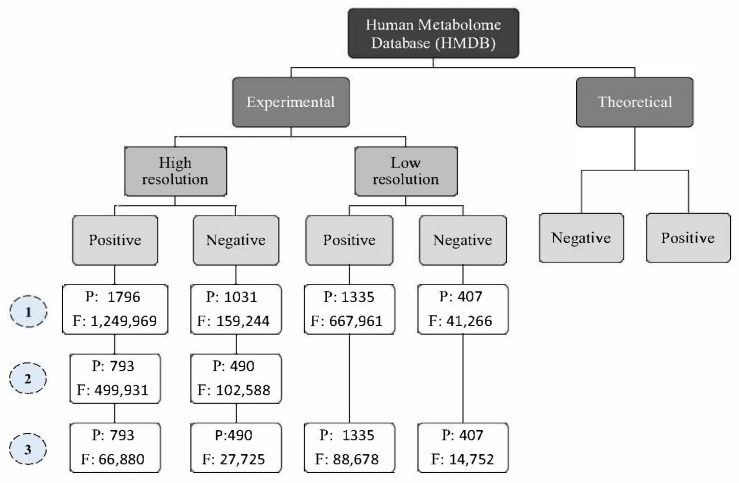
Human Metabolome Database (HMDB) classification. The sign No. (1) refers to the initially collected parents (P) and fragments (F). No. (2) shows the parents and fragments after removing the exogenous molecules. No. (3) represents the final parents and fragments database.

The current library was assembled by collecting MS1 and MS2 spectra of high-resolution experimental metabolites; 1282 for both positive and negative modes of 955 available unique compounds in the HMDB database using IDA acquisition mode (Figure 6 and Supplementary Tables S2A,B and S3A,B). The composite MS2 spectral library generation was used later for fragment data analysis.

### 2.5. Acquisition Method

We performed LC separation using a reversed-phase XSelect HSS T3 analytical column (Waters Co., Milford, CT, USA), which is compatible with a 100% aqueous mobile phase and provides ultra-low MS bleed while promoting polar compound retention. The acquisition was operated at two different pH values, 3.0 and 8.0, for positive and negative modes, respectively, to assemble the MS2 library. Coupling both modes increases the confidence of metabolite identification. Because some isomers are only separated under a specific condition, shuffling different chromatographic conditions provides additional confirmation of the compound’s existence when detected in both modes.

In the current work, the isolation window of the precursor ions for MS1 was 50–1100 Da, and the MS2 fragment ions scan was acquired with 50 Da intervals at a scan rate of 40 Hz. SWATH cycle was 0.05 s, and the scan rate ranged from 20 to 50 Hz to provide better coverage. Various implementations of the SWATH method have been described using different isolation windows ranging from wide to narrow *m*/*z* range depending on acquisition range and time [20]. At the same time, broader *m*/*z* SWATH windows increase the number of concurrently fragmented precursors, and the complexity of the acquired composite fragment ions spectra increases.

While performing SWATH scans, the link between fragment ions and precursors from which they originate is lost. Previous suggestions to link the transition ions with their precursor depend on manual chromatographic profile matching, which is not applicable, especially in the case of low resolution, poor peak shape, or various co-eluting molecules [12]. An alternate approach is to submit the raw samples to an online library with different search algorithms, which usually generate high false-positive identification due to wide-precursor windows [12].

The fragmentation pattern is robustly dependent on the instrument specification and collision energy settings [18]. In the current study, the precursor ion was accelerated using collision energy (C.E.) of ±35 eV and the C.E. spread ±20 eV based on widely accepted parameters observed in HMDB.

While most fragment production is maximized between 25 and 70 eV, at higher energy, the maximum fragments involve multiple bonds and ring cleavages, producing less intense signals [6]. On the other hand, using lower energy corresponds to neutral losses and single bond cleavages that yield more intense and clear signals. The HMDB library is built depending on a few discrete collision energies, such as 10, 20, and 40 eV serving as a base for generating unanimity spectra [8]. A high-resolution fragment database assists in developing high-resolution spectra acquired at discrete collision energies, which provide optimum precursor-fragment ion pairs and the corresponding collision energy [21]. The new technology offers a collision energy ramp to ensure appropriate fragmentation of most molecules. Previously, Bruderer et al. described a workflow to build high-quality mass spectral libraries for SWATH mass spectrometry data processing with discrete and composite collision energy values [8]. The authors found that the collision energy range was 10–70 eV in positive mode, which gave informative MS2.

### 2.6. Biological Samples Analysis

**Figure 7:**
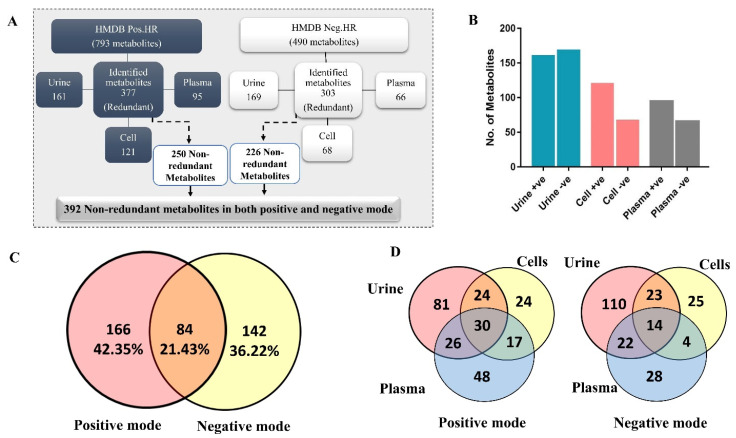
Biological sample analysis using the validated pipeline. (**A**,**B**) The identified molecules per biological sample. (**C**,**D**) The overlapping per mode or sample type.

We further validated the impact of the matrix effect on different biological samples using urine, plasma, and HL-60 cell line samples (Figure 2, Stage IV). An analysis of biological samples yielded 680 small molecules integrated into several pathways in the biological metabolomics processes. In positive mode, 377 small molecules were detected with different adducts ([M + H]^+^, [M + Na]^+^, [M + NH_4_], and [M + K]^+^), and 303 molecules in negative mode with adducts ([M − H]^−^, [M + Na − 2H]^−^, [M + NH_4_ − 2H], [M − Cl]^−^, [M + K − 2H]^−^, [M + FA − H]^−^, and [M + H_2_O − H]^−^). For both modes, 392 unique non-redundant metabolites were recorded. Supplementary Table S8 and Figure 7 summarize the numbers of identified compounds in different biological samples for both modes. These metabolites were further validated with their corresponding MS2 spectra. Of note, some precursors were acquired with different adducts, a condition that may mislead accurate identification, but that is not the case here, where the current platform could identify signal redundancy with different adducts that eliminates erratic identification [21]. The current workflow utilizes narrow precursor windows and strict filtration criteria that lead to limiting impurities and/or background masses.

In a previous experiment, Simon-Manso et al. performed qualitative metabolic profiling of a standard human plasma reference (SRM 1950) [8]. A total of 322 compounds were identified using four different LC-MS/MS platforms in positive mode. Most MS2 were recorded in IDA mode with a tolerance of 1.3 *m*/*z*. One of the drawbacks of this approach was the wide mass tolerance used, which might lead to false-positive identification. Another trial evaluated the relevance and versatility of LC-MS/MS using IDA on human serum metabolome using 657 representative metabolite standards [22]. The analysis was based on three stationary phases: a reversed-phase, a hydrophilic interaction chromatography, and a pentafluorophenyl propyl stationary phase for both positive and negative electrospray modes to achieve a high coverage rate. Despite the 266 metabolites identified, the approach was time-consuming and costly.

In the current study, plasma samples identified low compounds (Supplementary Table S8). This might be because plasma samples contain many non-polar compounds, which are lacking in HMDB [1]. In addition, we used a reversed stationary phase, a widely-used approach for chromatographic separations in the field of metabolomics. However, it might not be the best solution for non-polar compounds with a high molecular weight of over 1100 Da [22]. One probable solution is to use a stationary phase containing embedded non-polar groups. This consideration will be applied in further experimentation.

### 2.7. Pathway and Enrichment Analysis

**Figure 8:**
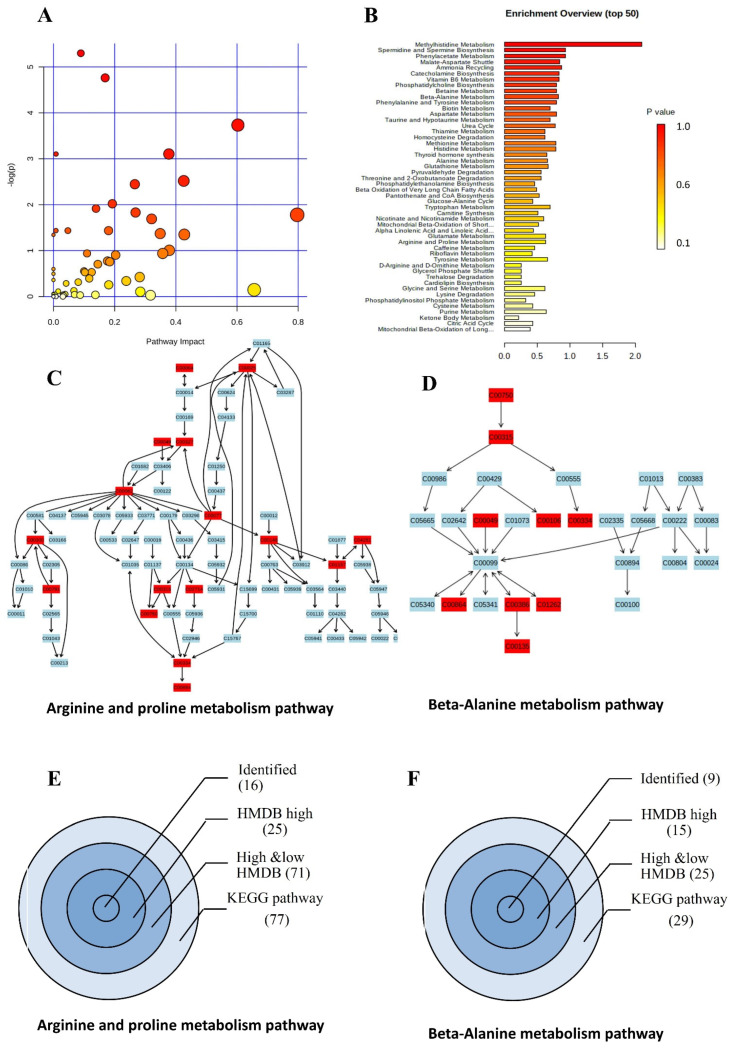
Pathway and enrichment analysis. (**A**) Pathway analysis of identified molecules. The node color recognizes the significance of the aligned molecule (*p* value). The node size correlates with the number of molecules identified within a given pathway. (**B**) The enrichment analysis of identified metabolites. Pathway color bars represent the *p*-value. Two pathways, beta-alanine metabolism (**C**) and arginine-proline (**D**) pathways represent in the KEGG database. Red boxes represent identified molecules within the current experiment. (**E**,**F**) Identified compounds of both molecules starting from the KEGG database.

The pathway analysis of 392 identified compounds involved 56 and 45 pathways from 90 and 98 in the KEGG and SMDB databases, respectively. These include the primary metabolic process in the biological samples (Supplementary Table S9, Figure 8). An enrichment analysis of these metabolites revealed 89 metabolic pathways (Supplementary Table S10, Figure 8). For example, we selected two highly covered pathways, “beta-alanine metabolism” and “arginine and proline metabolism” (Figure 8), to check the pathway coverage of the search.

The first glimpse revealed that our methodology identified between 20% and 30% of the molecules from the total number of molecules involved in a given KEGG pathway. Since 50–70% of molecules in KEGG pathways have no annotated pathway information, our analysis covered over 60% of high-resolution annotated KEGG molecules in a given pathway at a maximum (Figure 8) in the current biological samples.

### 2.8. Automation

A software tool was developed to analyze metabolomics samples based on our approach to overcome the overwhelming manual annotation, analysis, and possible mismatching errors. The semi-automated software, named Swath Auto System Analyzer (SASA), was successfully tested and validated manually and is freely available on (https://sourceforge.net/projects/sasatool/) accessed on 1 September 2022 with associated documentation and illustrative videos. The idea behind the software is to pick up and match the SWATH-acquired samples with the generated database. We implemented two major databases: HMDB for human biological samples, and ReSpect, a database for phytochemicals. A customized database based on the researcher’s preference is also an option in the software. SASA supports downstream analysis with a unique scoring system. The scoring utilizes several parameters, such as intensity ratios and retention times. The software generates pathway and enrichment analysis via KEGG with graphical output.

## 3. Materials and Methods

### 3.1. Chemicals and Standards

Acetonitrile, isopropanol, methanol, dichloromethane, and ethyl acetate were purchased from Thermo-Fisher (Thermo-Fisher Scientific, Waltham, MA, USA). Formic acid, ammonium hydroxide, ammonium formate, and ammonium acetate were purchased from Sigma-Aldrich (Sigma-Aldrich Co., St. Louis, MO, USA). Cell culturing media, penicillin-streptomycin, and fetal bovine serum were purchased from Gibco (Thermo Fisher Scientific, Waltham, MA, USA). Ten authentic standards were obtained from Sigma-Aldrich (Supplementary Table S1A). Standards set composed of vitamins and amino acids proposed in our pipeline for validating filtration criteria and SWATH platform. Stock concentrations (10 µg/mL) were prepared by dissolving analytes in methanol, except phenylalanine and folic acid were dissolved in a mixture of methanol 90% and water 10%. The working solution of each standard was prepared at a final concentration of 200 ng/mL in the same solvent as the stock solutions.

### 3.2. Filtration Criteria

We developed a novel data analysis platform (SASA) to analyze samples using the SWATH acquisition mode. The platform uses a high-resolution IDA spectral database available online (HMDB) to generate a DIA database based on fragment ions XICs. The rationale of the filtration criteria platform relies on extracting transition ions and aligning them with their precursor ions, using a priori information collected from the spectral library. The transition ions’ relative intensities within the IDA spectrum, chromatographic alignment, and their concurrent precursor ion were used to filter fragment ions’ XICs, which are precisely coherent with a targeted compound. An illustration of pipeline filtration criteria is shown in Figure 1. The leading five steps of the workflow include (1) building the library (MS1, MS2, and the relative intensity); (2) detection of the MS1 peaks; (3) detection and aligning XICs of MS2 with MS1; (4) filtration of the detected MS2 XICs; (5) visualization of the retrieved product ions with their precursor ions.

**Step 1** analysis aims at generating the database. The IDA databases were gathered from online workbenches, including the precursor and the product ions (fragments). The current work used a high-resolution human metabolome database (HMDB) (Supplementary Table S2A,B). A fragment’s relative intensities per component SPLASH (SPectraL hASH; database-independent identifier) were filtered and stratified depending on their relative intensity. Those with relative intensity higher than 75% were considered the main daughter (base peak) and tagged as (D), while fragments with relative intensities lower than 5% were excluded. Fragments >5% and <75% were considered secondary daughters and tagged as (F). This calculation was used to overcome the acquisition variability in the database [23]. Moreover, precursors not aligned with the main daughter (D) were ignored (Supplementary Table S3A,B).

**Step 2** assigns compounds using precursor mass and adduct species in designated samples. Next, samples and blanks were injected in SWATH acquisition mode. All precursor ions were searched against SWATH injected sample(s) using PeakView 2.2 with the MasterView 1.1 package (AB SCIEX, Framingham, MA, USA). MasterView was used to search for each precursor ion in different adduct formats based on predefined search criteria: precursor ion XIC signal-to-noise ratio (S/N) > 30, sample: blank > 5 with a precursor mass tolerance of 10 ppm. Adducts used for precursor formula search in positive mode were [M]^+^, [M + H]^+^, [M + Na]^+^, [M + NH_4_], and [M + K]^+^ and for negative mode were [M − H]^−^, [M + Na − 2H]^−^, [M + NH_4_ − 2H]^−^, [M − Cl]^−^, [M + K − 2H]^−^, [M + FA − H]^−^, and [M + H_2_O − H]^−^. A tailored list generated from MasterView of the candidate’s metabolites is used for subsequent steps.

**Step 3** detects XICs for each precursor ion and their fragments at the same SWATH window. Each candidate metabolite (Step 2) and its cognate fragment ions were retrieved from the database (Step 1). MultiQuant Software (AB SCIEX) was used to process samples and export data for each precursor and fragment XIC; peak height, retention time, and full width at half maximum (FWHM).

**Step 4** is designed to assign elemental compositions to the observed transition ions using the precursor mass and adduct species. The exported data were filtered depending on the parameters obtained from the validation method. Transition ions with height ratio in samples to blank as well as absolute (ABS) retention time (RT) shift and FWHM for each fragment from its precursor ion were kept. For each metabolite, the relative intensities were calculated, and XICs with relative intensity below 5% were neglected. Likewise, redundant XICs over different “SPLASHs” and precursors that remained without prominent daughters were neglected.

The remaining identified metabolites with a high degree of confidence were used for subsequent analysis using PeakView 2.2 with the SWATH software package (AB SCIEX, Framingham, MA, USA). Manual validation was performed to confirm the ideal alignment of fragment ions with their precursor and to give more credence to our approach, as shown in Section 2.

Calculated values were estimated based on the following equations:

1. The accepted retention time shift: where any retention time shift above this value is excluded.

$$RT\_Shift=abs\left(\frac{Parent\;RT-Fragment\;RT}{Parient\;RT}\right)$$

2. The accepted width shift: where any width shift above this number is excluded.

$$width\_Shift=abs\left(\frac{Parent\;width-Fragment\;width}{Parent\;width}\right)$$

### 3.3. Validation of Filtration Criteria

Validation of filtration criteria was conducted using two predefined lists of small molecules to determine the acceptable ABS retention time and FWHM shift limits and to estimate the false-positive and negative rates within the data.

The first list was a highly confident spectrum for ten standards (inclusion list) generated in IDA mode to construct an actual fragmentation spectral library (Supplementary Table S1A). The standard working solutions (200 ng/mL for each) were injected individually and in a multiplex format using both IDA and SWATH acquisition modes. XIC for each precursor ion in IDA mode was searched in the SWATH mode to estimate its RT and peak width shifts against the same metabolite precursor ion (Supplementary Table S4A,B). The standard deviation resulting from the total shift for all ions was used as the pipeline’s input criteria (Supplementary Table S4B).

The second list was the exclusion list, which includes 87 drugs from HMDB. The exclusion list consisted of 298 drugs, which later were searched against the KEGG database using MetaboAnalyst 4.0 to exclude molecules with endogenous origin (Supplementary Table S5A) [24]. This analysis returned 87 molecules with high confidence that should not be expressed within the cells and used as a negative control. The inclusion and exclusion lists were merged and used as a validation dataset to test the fidelity of previously explained pipeline filtration criteria (Supplementary Table S5B). The standard mixture was spiked in a biological sample (HL-60 cell line) and injected in a SWATH acquisition mode to check the filtration criteria and the matrix effect on the filtration pipeline (Figure 2; Stage II). The reason behind selecting HL-60 cells is to avoid any drug residue or exogenous metabolite that may exist in other biological samples (Plasma or urine).

### 3.4. Generation of Spectral Library and Assembling (IDA-DB Curation and DIA-DB Creation)

All metabolites’ precursor ions from HMDB, including serum, urine, saliva, feces, and cerebrospinal fluid samples, were collected and segregated into experimental and theoretical spectra. Next, the experimental spectra were sub-grouped into four categories: high-resolution positive mode, high-resolution negative mode, low-resolution positive mode, and low-resolution negative mode. The experimental high-resolution (HR) metabolites library (1282 metabolites for both positive and negative modes with 955 unique compounds; Supplementary Tables S2A and S3B) with their HR fragments in both positive and negative modes were merged as illustrated in Supplementary Table S3A,B. The tailored database was used to detect metabolites in the biological samples (Figure 2; Stage III). Finally, the DIA database was created, and annotated accurate fragments of ions were constructed.

### 3.5. Samples Extraction Protocols

The feasibility of our built-in library was tested within biological samples, and the effect of sample complexity on transition ion extraction was examined. Three samples were used; HL-60 cell line, plasma, and urine. The extraction solvent used for metabolites extraction consisted of methanol: dichloromethane: ethyl acetate: acetone 1:2:3:6, respectively. Reconstitution solvent consisted of water: methanol: acetonitrile 2:1:1, respectively.

#### 3.5.1. Cell Line Preparation

The human leukemia cell line (HL-60) was seeded in RPMI 1640 (Roswell Park Memorial Institute, Buffalo, NY, USA) supplemented with 1% penicillin-streptomycin and 20% fetal bovine serum. The media with its supplements was changed after three days for nutrient replenishment. Cell line doubling time was 36–48 h after incubation at 37 °C with 5% CO_2_. Cell count and viability were determined using trypan blue using a microscope counting chamber.

Plates containing 5 × 10^5^ cells/plate were harvested on ice and centrifuged at 3000 rpm for 3 min at 4 °C. The pellets were washed with 300 µL precooled (−80 °C) PBS, centrifuged, and the pellet was stored at −80 °C until extraction [25]. The pellets were thawed on ice, and 600 μL from the precooled extraction solvent was added, followed by vigorous shaking for 2 min and sonicated for 5 min at 30 kHz. The samples were exposed for three freeze–thaw cycles to ensure maximum extraction of the small molecules [26] and centrifuged at 10,000 rpm for 10 min at 4 °C. The supernatant was separated into new tubes and evaporated using a vacuum rotary evaporator. Dried samples were reconstituted in 200 µL reconstitution solvent, sonicated, centrifuged, and finally, the supernatant was transferred to analysis vials for mass spectrometric analysis.

#### 3.5.2. Plasma and Urine Preparation

Plasma and urine samples were obtained from voluntary healthy donors and stored immediately at −80 °C. Upon analysis, samples were thawed on ice, and aliquots of 200 μL were taken in glass tubes. A total of 800 μL of a precooled extraction solvent was added, followed by vigorous shaking for at least 2 min. The samples were processed as mentioned before. The extracted samples were injected in positive and negative modes using SWATH acquisition.

### 3.6. SWATH and IDA Acquisition Methods

Metabolite separation was carried out using a SCIEX EXION LC^TM^ AC UHPLC system using a Phenomenex In-Line filter disk 0.5 µm × 3.0 mm equipped with Acquity XSelect HSS T3 analytical column 2.1 × 150 mm, 2.5 µm I.D. maintained at 40 °C (Waters Co., Milford, CT, USA). A 28 min gradient elution with a flow rate of 300 µL/min was used. The mobile phase solutions were as follows, mobile phase solution (A); 5 mM ammonium formate in 1% methanol (pH 3.0) for positive mode, solution (B); acetonitrile, and solution (C); 5 mM ammonium formate in 1% methanol (pH 8.0) for negative mode elution. Gradient elution was sustained at 0% B for 1 min, 0% B to 90% B in 20 min, 90% for 4 min, 90% B to 0% B in 0.1 min, and finally re-equilibrating with 0% B for 3 min.

Mass spectrometric analysis was performed using a Triple TOF^TM^ 5600^+^ system equipped with a Duo-Spray^TM^ source operated in the ESI mode (AB SCIEX: Concord, ON, Canada). The ions spray voltage capillary was 4500 eV with declustering potential voltages of 80 V in positive mode and −80 V in negative mode. The source temperature was set at 600 °C, the curtain gas was 25 psi, and gas 1 and 2 were 40 psi. The collision energies were 35 V in positive mode and −35 V in negative mode with CE spreading 20 V. TripleTOF 5600 was operated using an information-dependent acquisition (IDA) and Sequential windowed acquisition of all theoretical MS (SWATH) methods. Batches for M.S. and MS/MS data collection were created using Analyst TF 1.7.1. Mass calibration was automatically performed at every 2 h analysis by the automated calibration delivery system using the APCI calibration solution (AB SCIEX).

#### 3.6.1. SWATH Acquisition

The SWATH acquisition method consisted of a single TOF scan from 50 to 1100 Da accumulated in 30 ms followed by a product ions TOF scan from 50 to 1100 Da using fixed 50 Da transition windows.

#### 3.6.2. IDA Injection

The IDA Acquisition method consisted of a single TOF scan from 50 to 1100 Da accumulated in 30 ms followed by 15 product ion TOF scans from 50 to 1100 Da for the most intense precursors, which exceed 200 counts per second (cps). Dynamic background subtraction was activated, and precursor ion selection was ignored for 3 s after three successive occurrences.

### 3.7. Pathway and Enrichment Analysis 

The identified metabolites in each biological sample underwent pathway and enrichment analysis using MetaboAnalyst 4.0 to investigate the integration of identified HMDB experimental high-resolution small molecules in the KEGG pathway [27].

### 3.8. Automation of the Pipeline (SASA)

The SWATH-Auto System Analyzer Tool, SASA Tool, was developed using python 3.7 [28]. The software is designed to run as an executable file on several windows platforms (detailed compatibility is fully described in the software documentation). Each function in the script was made to run on a separate thread. Numpy and pandas arrays were used to increase the speed and efficiency of processing the data. The software process workflow consists of 4 main steps: (1) database selection step, where the user selects or/and generates a customized database; (2) parents detection step using MasterView, where SASA speeds up the search through the generation of the MasterView method; (3) fragments detection step using MultiQuant, where SASA calculates the start and end masses and experimental window and generates the MultiQuant method; (4) parents and fragments filtration, where the filtration process is applied using accepted predetermined parameters. In the current biological samples analysis, ions with height ratios in samples to blank were ≥5, ABS retention time (RT) shift for each fragment from the precursor ion was <0.1417%, and FWHM for each fragment from the precursor ion was <17.4965.

## 4. Conclusions

Refining and using metabolomics database libraries such as HMDB or Metlin as curated public databases enable maximum efficiency of correct metabolite fragment matching. This matching could be operated via unbiased SWATH analysis by matching precursor ions with their possible transition signals. Integrating SASA algorithms and scoring systems minimizes mismatching and lowers the potential for possible false hits. An emerging approach toward precision medicine is beneficial for assessing biological system disturbances resulting from diseases.

## Data Availability

SASA is freely available on SourceForge (https://sourceforge.net/projects/sasatool/ accessed on 1 September 2022) with complete documentation and tutorials (https://www.57357.org/app/uploads/2019/12/SASA-documentation-1.pdf accessed on 1 September 2022) see the Supplementary pdf termed 1. SASA-documentation-1. The SASA code is open access and available on: (https://github.com/Proteomicslab57357/SASA accessed on 1 September 2022). Demo Files: https://github.com/Proteomicslab57357/SASA accessed on 1 September 2022. The metabolomics generated data are available via “MassIVE” repository with study identifier [project ID: MSV000090242], link https://massive.ucsd.edu/ProteoSAFe/dataset.jsptask=b7a30f9902374854bc2a6d8d4cde9493 accessed on 1 September 2022, ftp://massive.ucsd.edu/MSV000090242/ accessed on 1 September 2022.

## Supplementary Materials

The following supporting information can be downloaded at: https://www.mdpi.com/article/10.3390/ijms231810908/s1.
