# Supplementary material for: A Novel Approach of SWATH-Based Metabolomics Analysis Using the Human Metabolome Database Spectral Library
Source: Int J Mol Sci. 2022 Sep 18;23(18):10908. doi: 10.3390/ijms231810908 (PMC9500730; doi:10.3390/ijms231810908)
Supplement: Supplementary file 1 [file ijms-23-10908-s001.zip › 1. SASA-documentation-1.pdf]

SWATH-Auto System Analyzer Tool (SASA Tool)

Tutorial

Table of Contents

Introduction 2
Application description 3
How to Download and Install SASA 5
The Start Window 6
The Main Window 7
Equations 15
MasterView 16
MultiQuant 22
Built-in Database 26
Adducts 27
Errors 29
SOFA 29
KEEG query 29
Citation 30

1. Introduction

SWATH-Auto System Analyzer Tool, SASA Tool (Version 1.0.0), is a novel SWATH platform for non-targeted metabolomics data analysis with accurate mass spectral library for metabolite identification using SWATH acquisition mode. Data analysis is based on scoring the alignment probability of certain precursor with its possible transition signals. This strategy is operated via unbiased SWATH based approach, in cyclic recording, over the acquisition time range of sequential survey scans and precursors transition ion spectra in predetermined isolation windows. The validated platform utilizes mass spectral library of high-resolution small molecules parsed from the HMDB for both positive and negative modes. The platform is well tested and validated manually using authentic standards and exclusion list. The fidelity of the workflow and the applicability of the library were tested using various biological samples; plasma, urine, and cell lines. SASA simplifies our pipeline algorithm in a semi- automated fashion and freely available on (https://sourceforge.net/projects/sasatool/). The software was developed using python 3.7. SASA is designed to be run as an executable file on several windows platforms. Each function of the script was made to run on a separate thread. Numpy and Pandas arrays were used to provide speed up, and efficient processing for the data. The software process workflow consists of 4 main steps; 1) Database choice step; where the user has to select and/or generate a database. 2) Parents detection step using MasterView; SASA speeds up the search through generation MasterView method, 3) Fragments detection step using MultiQuant; SASA will calculated the start and end masses, experimental window, and generate MultiQuant method , 4) Parents and fragments filtration; final step where the filtration process is applied using accepted predetermined parameters. In the current biological samples analysis, ions with height ratio in samples to blank was ≥ 5, absolute (ABS) retention time (RT) shift for each fragment from the precursor ion was $< 0.1417\%$, and ABS peak width at half height for each fragment from the precusror ion was <17.4965. Our future prospective is to upgrade SASA into a fully automated standalone software with integration of various comprehensive databases.

Project PI: Sameh Magdeldin, Proteomics and Metabolomics Unit, Department of Basic Research, Children's Cancer Hospital 57357, Sameh.Magdeldin@57357.org

Lead developer: Ali Mostafa, aliali.mostafa99@gmail.com, Cairo University.

Main contributors: Eman Ahmed, Proteomics and Metabolomics Unit, Department of Basic Research, Children's Cancer Hospital Egypt 57357. Eman.abdelnaby@57357.org

2. Description

The software process workflow consists of 4 main steps and each step contains various sub-steps;

1- The database choice step, where the user has to select or/and generate a database.

2- Parents detection step using MasterView (PeakView 2.2 with the MasterView 1.1 package (AB SCIEX), the software will save a lot of time generating the method for the MasterView by one click.

3- Fragments Detection step using MultiQuant software (AB SCIEX), the software will save a lot of time generating the method for the MultiQuant by one click.

4- Parents and Fragments Filtration, this is the final step where the filtration process is applied using accepted shifts entered by the user.

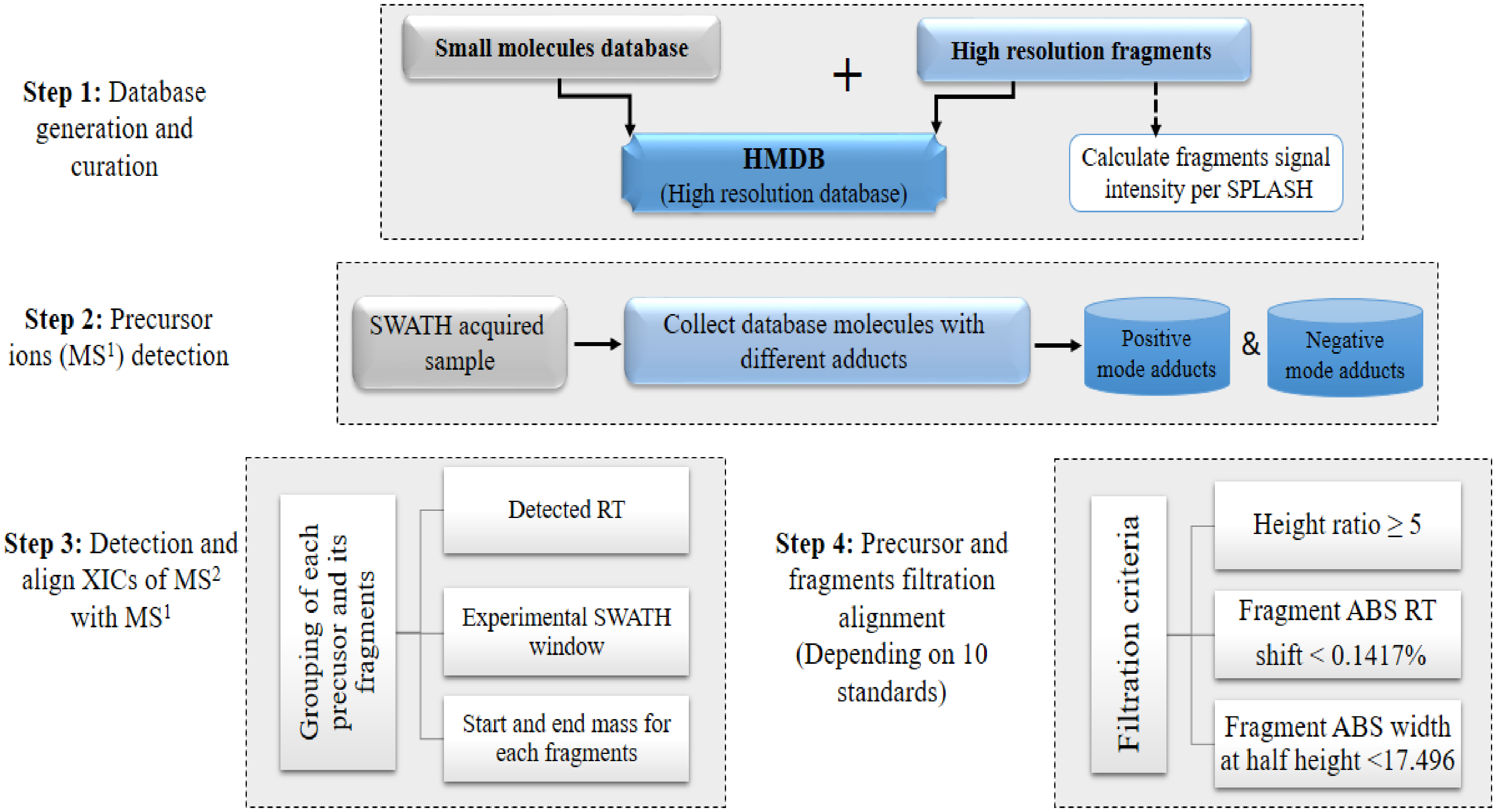

Fig.1 Schematic illustration of SWATH-Auto System Analyzer (SASA) workflow for small molecule extraction of SWATH acquired samples.

3. How to Download and Install SASA

1. Download SASA tool, which can be downloaded from https://sourceforge.net/projects/sasatool/
2. Extract the file named SASA
3. Run the Installation from the installation icon.
4. This screen will pop up.

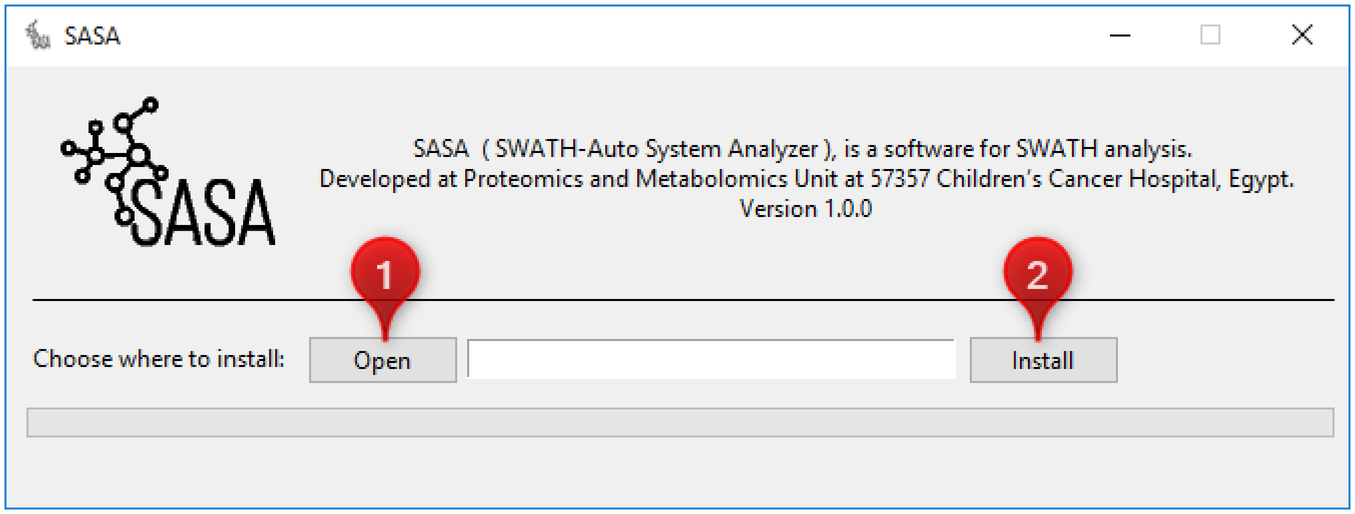

Fig.2: SASA installation panel

5. Choose installing extension of SASA from button 1 (Open).
6. Press button 2 (Install).
7. A SASA shortcut icon will be created on the desktop. Now you can run the SASA tool from this icon.

4. GUI description

The GUI of the tool is friendly user interface. The GUI of SASA tool consists of two windows; start window and main window.

4.1. The Start Window

It contains six buttons:

1- The first one named “About” and by clicking it, a summary describes of SASA Tool will be displayed for the user.
2- The second one named “Open documentation file”, by clicking this button, the pdf tutorial file will be opened.
3- The third button called “1. Check requirements”, since the SASA tool depends on two other software; MasterView and MultiQuant, therefore, this button will check for both software either they are installed on the C drive or not. If not available on the C drive, the user has to set the paths that define the location of both software.
4- “2. Choose Output Folder” button allow the user to choose which folder that the results will be saved on.
5- The last one “3. Start the software” will take the user from the start window to the main window.

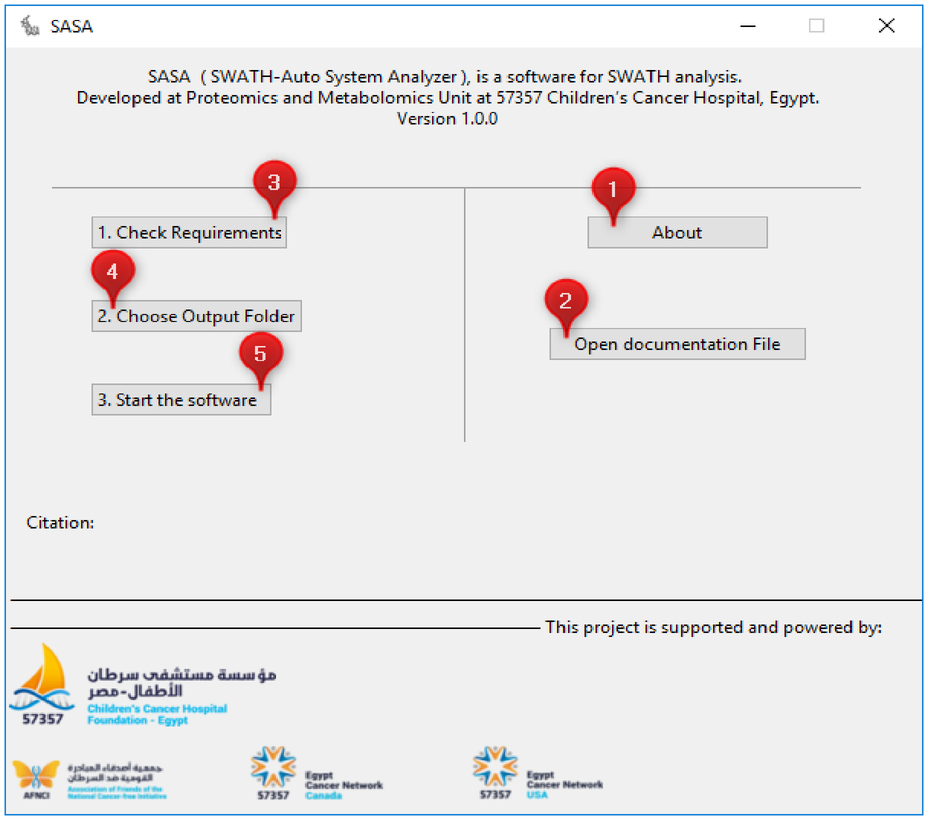

Fig.3: SASA start window

Note: Before starting the application you should see two true signs.

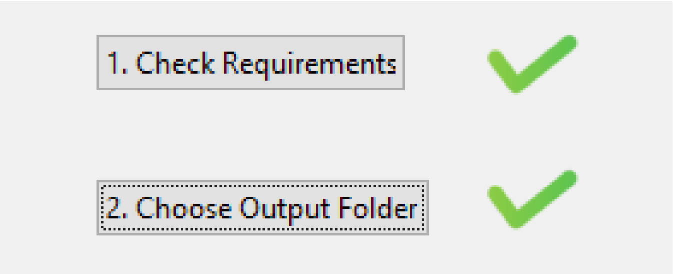

Fig.4: Requirements and output path availability signs

4.2. The Main Window

1- Toolbar: contains 3 objects “File”, “Converter”, and “Tools”.

a- “File” contains other 3 objects; “Open” to open any result file, “Start new sessions” to restart the program and start a new project, and “Quite” to close the program.

b- “Converter” it is an additional option provided by SASA to convert any metabolomics file format to another for example convert Wiff to MZxml.

c- “Tools” contains 2 objects “Open SOFA” where SOFA is an open statistics software (for more information: https://www.sofastatistics.com/downloads.php), and “KEGG query” where the user can produce a hit graph from KEGG database (more information in “KEGG query” section).

2- Step number 1, described in details in “GUI steps” section.

3- Step number 2, described in details in “GUI steps” section.

4- Step number 3, described in details in “GUI steps” section.

5- Step number 4, described in details in “GUI steps” section.

6- Progress bar.

7- History window where any process done by the user is recorded.

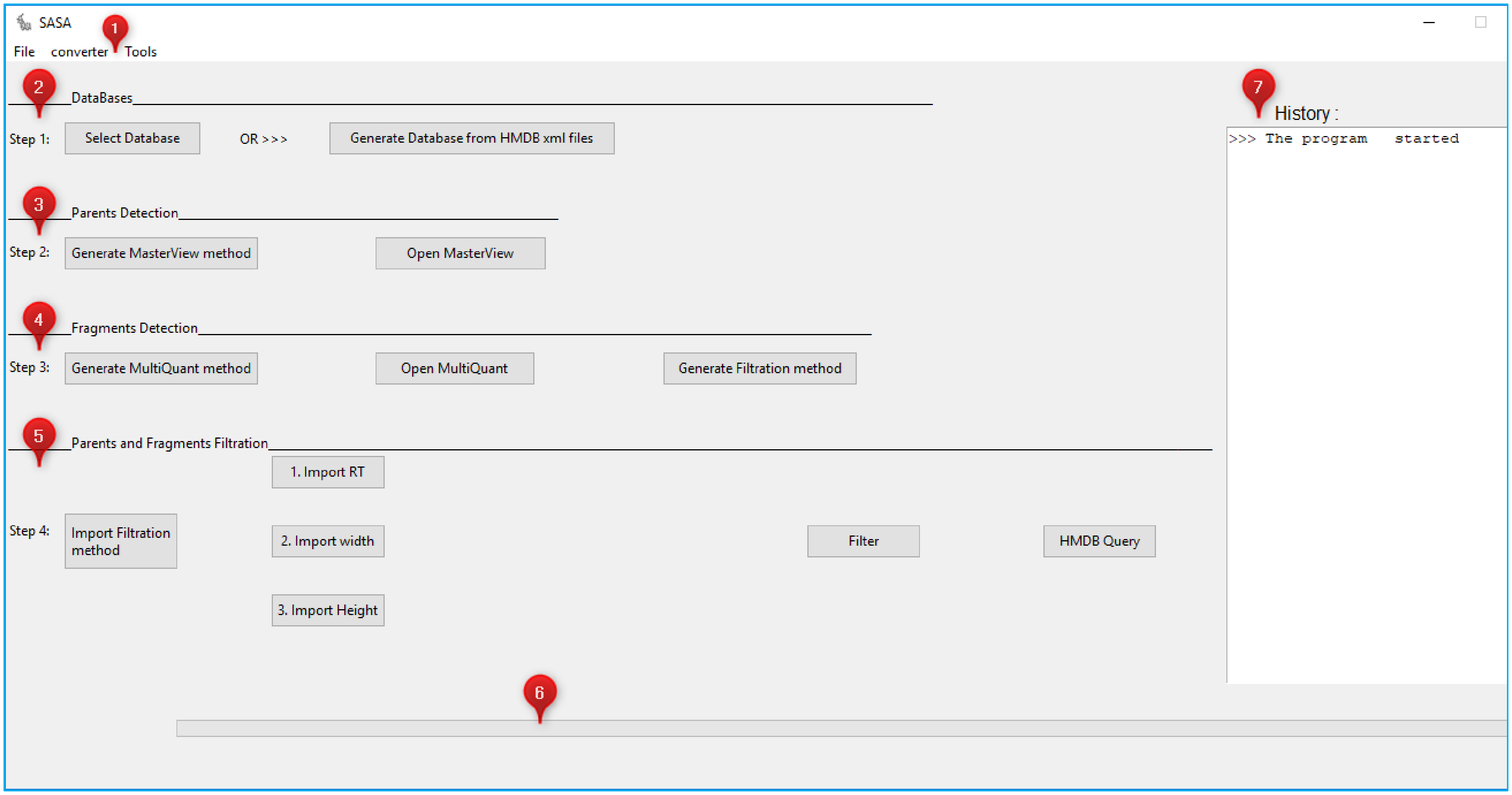

Fig.5: SASA main window

5. GUI steps

5.1. DataBase (First Step).

The user can choose between three options;

1- To select from built-in SASA tool databases. For more details about the built-in databases see section (Built-in Database).

2- To generate desired metabolites list from Human Metabolome Database (HMDB) XML format to csv where the SASA tool can use (customized based on user preference).

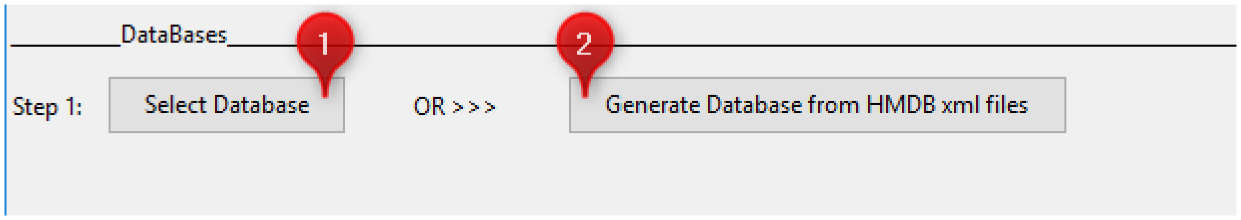

Fig.6: SASA DataBase (first step) options

- Parameters needed in this step;

  No parameters

- The expected results from this step;

  If the user chooses to make his own Database from option 2 or 3, the resulted file will be a CSV file for the selected metabolites list. This step is a prerequisite because SASA tool expected the database in a certain CSV format (You will find file example in the demo section).

5.2. Parents Detection (Second step).

There are 2 sub-steps in the second step;

1- Generate a method for MasterView software using all available adducts depending on the acquisition mode either positive or negative mode.
2- Parent's detection using MasterView software. The user have to open MasterView software to run this analysis. In this sub-step, we expect from the user to export the results from the MasterView to be used in the next step. For more Details on how to use MasterView see "MasterView" section.

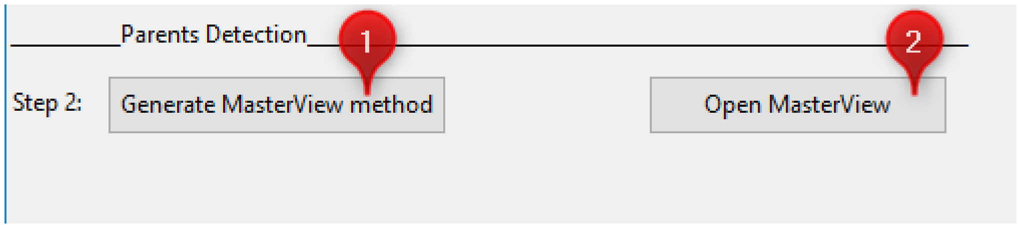

Fig.6: SASA Parents Detection (second step) panel

➢ Parameters and files needed in this step;

1- For MasterView output generation SASA need only one parameter termed "width (Da)" [0.001-0.01Da].
2- The Adducts file (See the Adducts section)

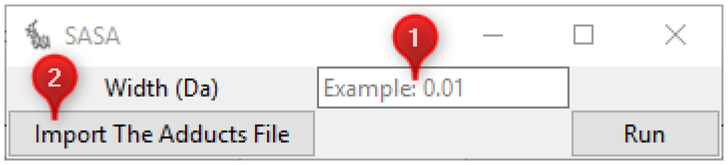

Fig.7: Parents detection (second step) sub-steps panel

➢ The expected results from this step;

1- The MasterView method from sub-step 2.1, which will be used in the next 2.2 sub-step. This file will be called “MVmethod.csv” (You will find file example in the demo section).

2- The user has to export a csv file from MasterView. For more details on how to use MasterView see “MasterView” section (You will find file example in the demo section).

5.3. Fragments Detection (Third step)

There are 3 sub-steps in the third step.

1- Generate a method for MultiQuant software.

2- Fragments detection using MultiQuant software. Open MultiQuant software to run the analysis. In this sub-step, we expect from the user to export the results from the MultiQuant to be used in the next step. For more details on how to use MultiQuant see “MultiQuant” section.

3- Generate filtration method, this method will retrieve each group of fragments for their precursor ion. The exported file will be used in the last step.

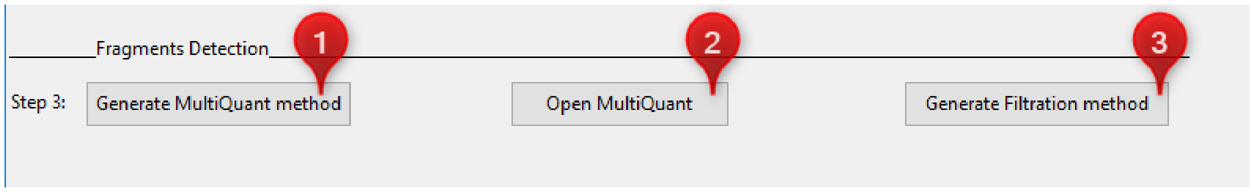

Fig.8: Fragment detection (third step) panel

➢ Parameters and files needed in this step;

1- For MultiQuant method, there are 5 parameters needed:

a- m/z shift, this number will be used from equation number 1 to calculate the “start” and “end” mass for each metabolite. (see Equations section)

b- Start m/z range window (see Equations section).

c- End m/z range window (see Equations section).

d- m/z swath window.

e- Import the MasterView.csv file resulted from MasterView analysis in sub-step 2.2.

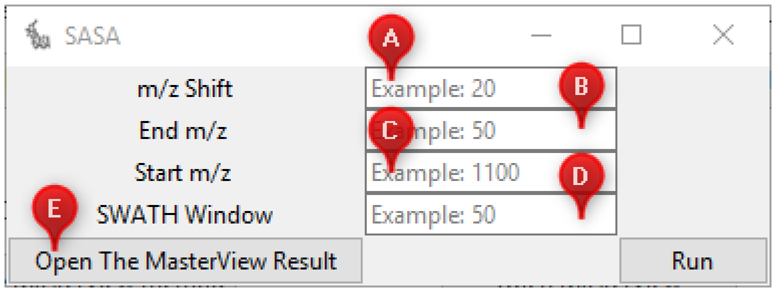

Fig.9: Parameter setting for generation MultiQuant method

2- For the Filtration method, only one parameter needed to be selected acquisition mode (positive ions or negative ions). Also, the File named “MQ_Filteration_method” should be imported.

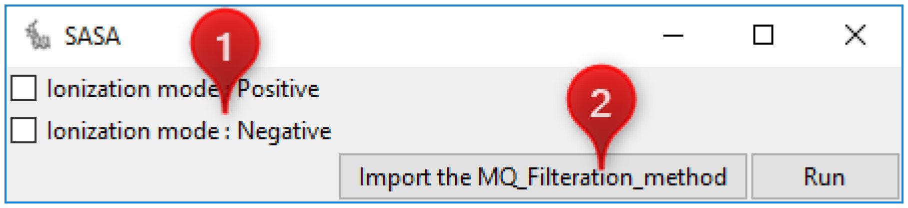

Fig.10: Parameter setting for generation Filtration method

➢ The expected Results from this step.

1- From sub-step 3.1 two txt files will be generated the first one named “MQmethod” this file will be used in sub-step number 3.2, the second file termed “MQ_Filteration_method” this file will be used in sub-step 3.3 (A file example in the demo section).

2- From sub-step 3.2, we expect from the user to export 3 files from MultiQuant (Retention time, Width at 50%, and Height). For more details on how to use MultiQuant see “MultiQuant” section (A file example in the demo section).

3- The last sub-step 3.3, the expected result from SASA tool is an excel file named “Filtration_method” which will be used in the final step (A file example in the demo section).

5.4. Filtration (Fourth step)

There are five sub-steps in this step;

1- Import the filtration method file "Filtration_method".

2- Insert the "retention time" file resulted from sub-step 3.2. After that, RT insertion, press "Run" retention time.

3- Insert the "width at 50%" file resulted from sub-step 3.2. Then, "Run" width at 50%.

4- Insert the "height" file resulted from sub-step 3.2.

5- Insert a number to add on the blank, to calculate the ration (See equation number 2).

6- Run Ratio.

8- Finally, press "filter" process.

9- Since Human Metabolome Database spectral xml file has no other information rather than the accessions and the spectral data, therefore, SASA tool is programed to overcome this problem issue by retrieving each accession information (Name, Formula, and Biological location) and return an csv file named "Final Result Information". Note this step will work only if the used database is HMDB (current version).

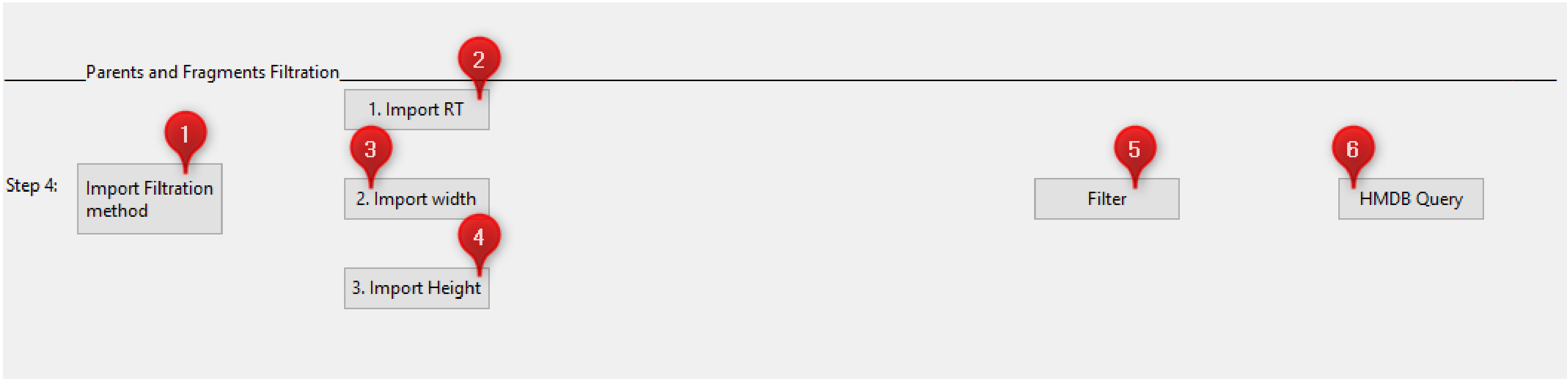

Fig.11: parents and fragments filtration (final step) panel

➢ Parameters needed in this step;

1- The parameters need in this step only required in sub-step 8.5.4 represent three Main criteria SASA tool make the filtration according to them;

a- The Accepted retention time, where any retention time shift more than this value will be deleted (See equation number 3).

b- The Accepted width, where any width shift more than this value will be deleted (See equation number 4).

c- The Accepted height ratio, where any ratio less than this value will be deleted (See equation number 2).

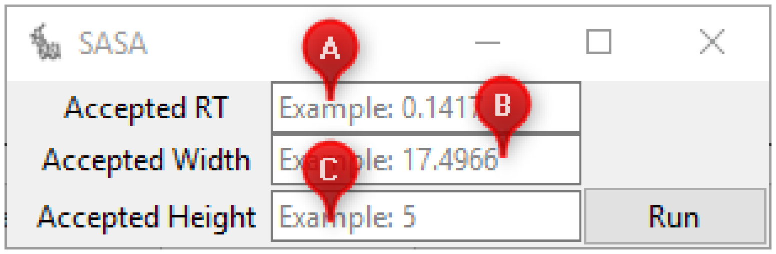

Fig.12: window of the accepted range with suggestion

- The expected results from each step.

1- The expected result from step 8.5.4 excel file represent the metabolites detected after filtration. The file entitled “Final Result” (You will find file example in the demo section).

Note: SASA give a score for each fragment according to how much a fragment retention time and width at 50% deviate from its parent. The scoring is in range of 0 to 1, where 1 or near to 1 mean that the fragment has a good alignment with the parent, and 0 or near to 0 mean that the fragment deviates from the parent.

2- The expected result from step 9.5.4; file named “HMDB”, contains (identified metabolite IDs, Name, Formula, Biological location, avr_RT_sample, avr_width_sample, Transition level (Sympol), and scoring %)

Note: the total scoring% here is different from the one mentioned above. Here the scoring is calculated for all fragments for a parent, to give information about the fragments alignments with the parents. It ranges from 0 to 100, approaching 100 mean the total fragments have good alignment with their parent, and 0 or near to 0 mean that the fragment deviates from the parent.

6. Equations

1- The m/z shift is used to calculate the ppm for the start m/z and the end m/z. The equation used:

$$Start\ mass = mz - \left(\frac{mz}{10^6} \times mz\right)$$

$$End\ mass = mz + \left(\frac{mz}{10^6} \times mz\right)$$

2- This equation used to calculate the ratio of a sample to a blank, where x is inserted by the user.

$$\frac{Height}{Blank + x}$$

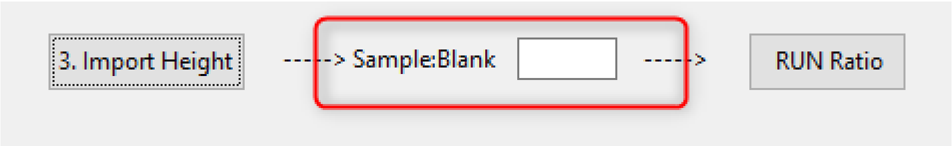

3- The Accepted retention time shift, where any retention time-shift above this number will be excluded.

$$RT\_Shift = abs\left(\frac{Parient\ RT - Fragment\ RT}{Parient\ RT}\right)$$

4- The Accepted width at half height shift, where any width-shift above this number will be excluded.

$$width\_Shift = abs\left(\frac{Parient\ width - Fragment\ width}{Parient\ width}\right)$$

5- The total scoring% for each parent was calculated as:

First we calculated a scoring for each fragment to its parent

$$fragment\ \ width\ scoring = |parent\ width\ at\ 50\% - \ fragment\ width\ at\ 50\%|$$

$$fragment\ \ retention\ time\ scoring = |parent\ retention\ time - \ fragment\ retention\ time|$$

To Account for the width at 50% and the retention time together:

$$fragment\ \ width\ and\ RT\ scoring = \ fragment\ \ width\ scoring + \ fragment\ \ retention\ time\ scoring$$

Then the result was normalized to be ranged from 0 to 1:

$$normalized\ fragment\ \ width\ and\ RT\ scoring\ \left|\frac{fragment\ \ width\ and\ RT\ scoring - min(fragment\ \ width\ and\ RT\ scoring)}{(max(fragment\ \ width\ and\ RT\ scoring) - min(fragment\ \ width\ and\ RT\ scoring))} - 1\right|$$

Finally, the total scoring% for each parent calculated as:

$$\left(\sum \frac{normalized\ fragment\ \ width\ and\ RT\ scoring}{count\ of\ \ fragments}\right) \times 100$$

7. MasterView

1- Open MasterView.

2- From the tool bar select MasterView then select new Session.

3- Import desired sample and blank.

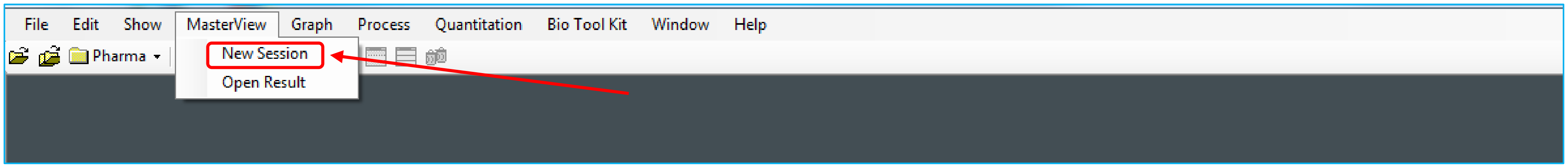

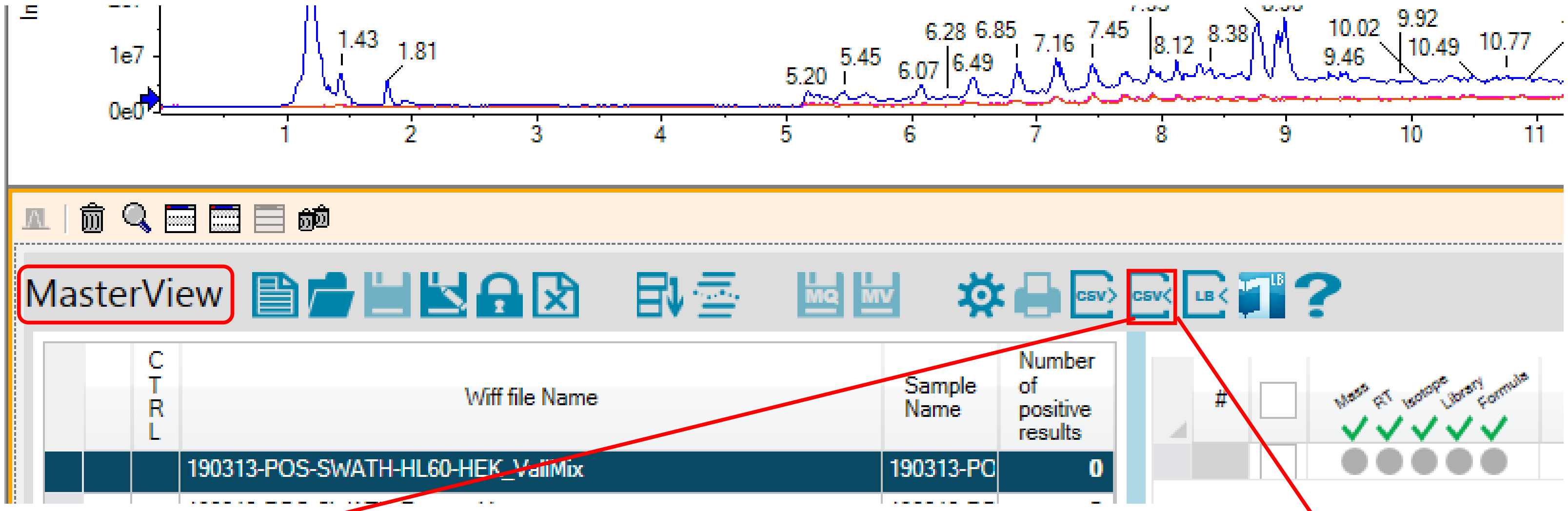

Fig.13: MasterView samples upload window

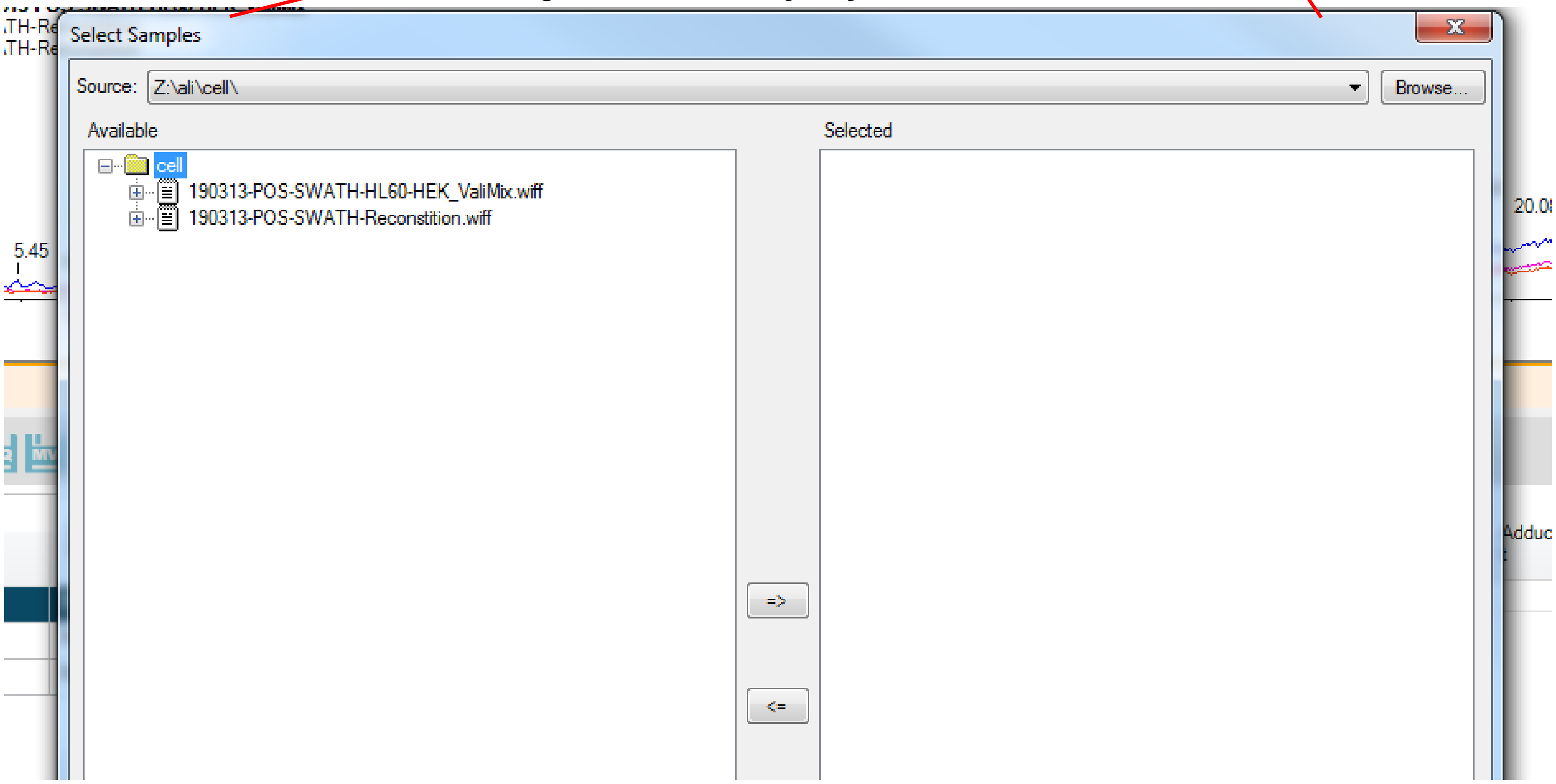

4- In the second half of the software titled MasterView, select “Import Compounds Info from CSV file” icon then import the MasterView method resulted from SASA tool. In the new window, press “Browse” and open the method that generated by SASA.

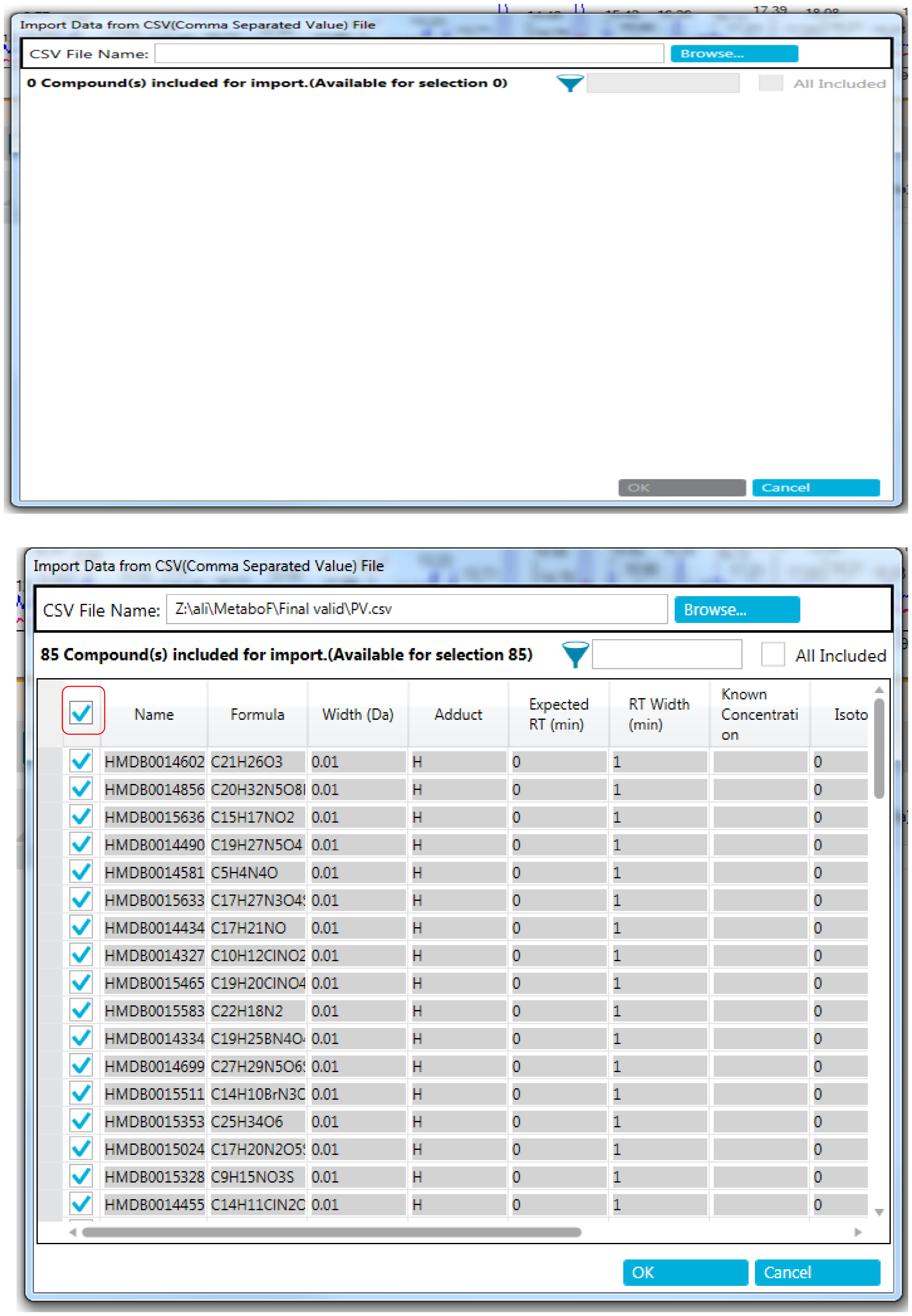

Fig. 14. MasterView parents database upload window

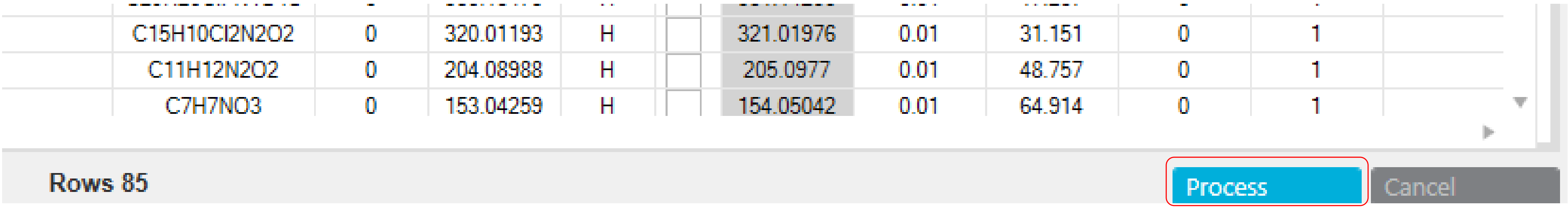

5- Then press Process.

6- Wait until the MasterView finish the process.

7- Wait until the MasterView finish the process.

8- Selec only the parents detected (highlighted by green light) and click the index box

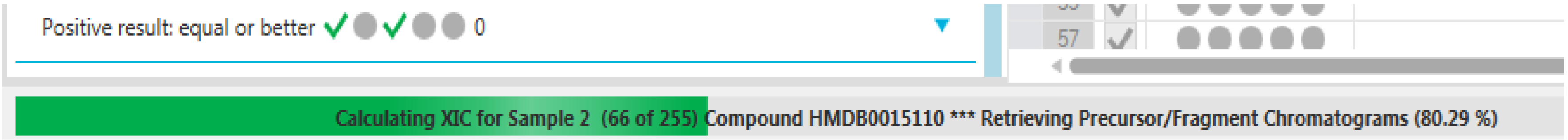

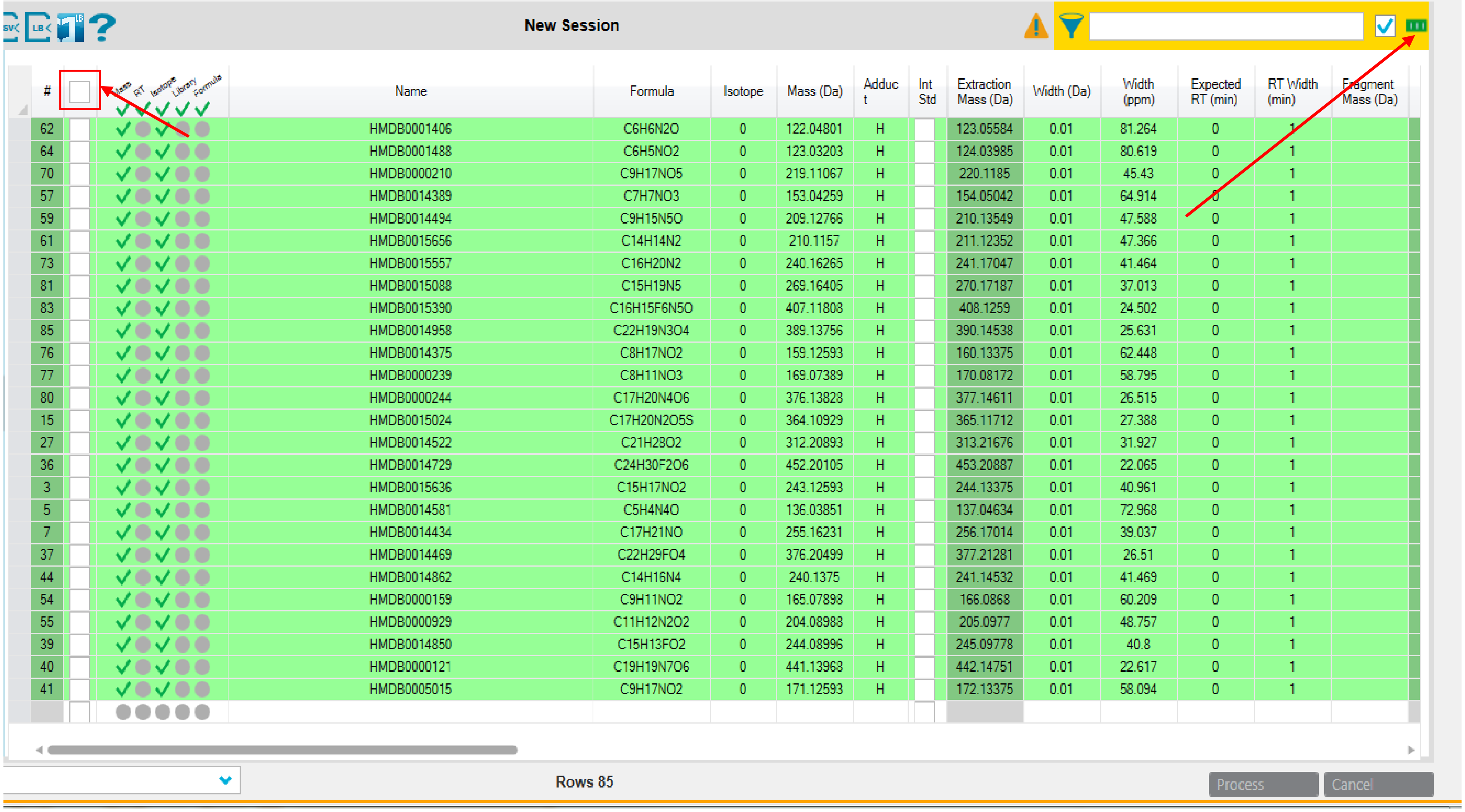

#	Name	Formula	Isotope	Mass (Da)	Adduct	Int Std	Extraction Mass (Da)	Width (Da)	Width (ppm)	Expected RT (min)	RT Width (min)	Fragment Mass (Da)
62	HMDB0001406	C6H6N2O	0	122.04801	H		123.05584	0.01	81.264	0	1	
64	HMDB0001488	C6H5NO2	0	123.03203	H		124.03985	0.01	80.619	0	1	
70	HMDB0000210	C9H17NO5	0	219.11067	H		220.1185	0.01	45.43	0	1	
57	HMDB0014389	C7H7NO3	0	153.04259	H		154.05042	0.01	64.914	0	1	
59	HMDB0014494	C9H15N5O	0	209.12766	H		210.13549	0.01	47.588	0	1	
61	HMDB0015656	C14H14N2	0	210.1157	H		211.12352	0.01	47.366	0	1	
73	HMDB0015557	C16H20N2	0	240.16265	H		241.17047	0.01	41.464	0	1	
81	HMDB0015088	C15H19N5	0	269.16405	H		270.17187	0.01	37.013	0	1	
83	HMDB0015390	C16H15F6N5O	0	407.11808	H		408.1259	0.01	24.502	0	1	
85	HMDB0014958	C22H19N3O4	0	389.13756	H		390.14538	0.01	25.631	0	1	
76	HMDB0014375	C8H17NO2	0	159.12593	H		160.13375	0.01	62.448	0	1	
77	HMDB0000239	C8H11NO3	0	169.07389	H		170.08172	0.01	58.795	0	1	
80	HMDB0000244	C17H20N4O6	0	376.13828	H		377.14611	0.01	26.515	0	1	
15	HMDB0015024	C17H20N2O5S	0	364.10929	H		365.11712	0.01	27.388	0	1	
27	HMDB0014522	C21H28O2	0	312.20893	H		313.21676	0.01	31.927	0	1	
36	HMDB0014729	C24H30F2O6	0	452.20105	H		453.20887	0.01	22.065	0	1	
3	HMDB0015636	C15H17NO2	0	243.12593	H		244.13375	0.01	40.961	0	1	
5	HMDB0014581	C5H4N4O	0	136.03851	H		137.04634	0.01	72.968	0	1	
7	HMDB0014434	C17H21NO	0	255.16231	H		256.17014	0.01	39.037	0	1	
37	HMDB0014469	C22H29FO4	0	376.20499	H		377.21281	0.01	26.51	0	1	
44	HMDB0014862	C14H16N4	0	240.1375	H		241.14532	0.01	41.469	0	1	
54	HMDB0000159	C9H11NO2	0	165.07898	H		166.0868	0.01	60.209	0	1	
55	HMDB0000929	C11H12N2O2	0	204.08988	H		205.0977	0.01	48.757	0	1	
39	HMDB0014850	C15H13FO2	0	244.08996	H		245.09778	0.01	40.8	0	1	
40	HMDB0000121	C19H19N7O6	0	441.13968	H		442.14751	0.01	22.617	0	1	
41	HMDB0005015	C9H17NO2	0	171.12593	H		172.13375	0.01	58.094	0	1	

Fig. 15. Aligned parents in the tested sample

9- Finally Export the results.

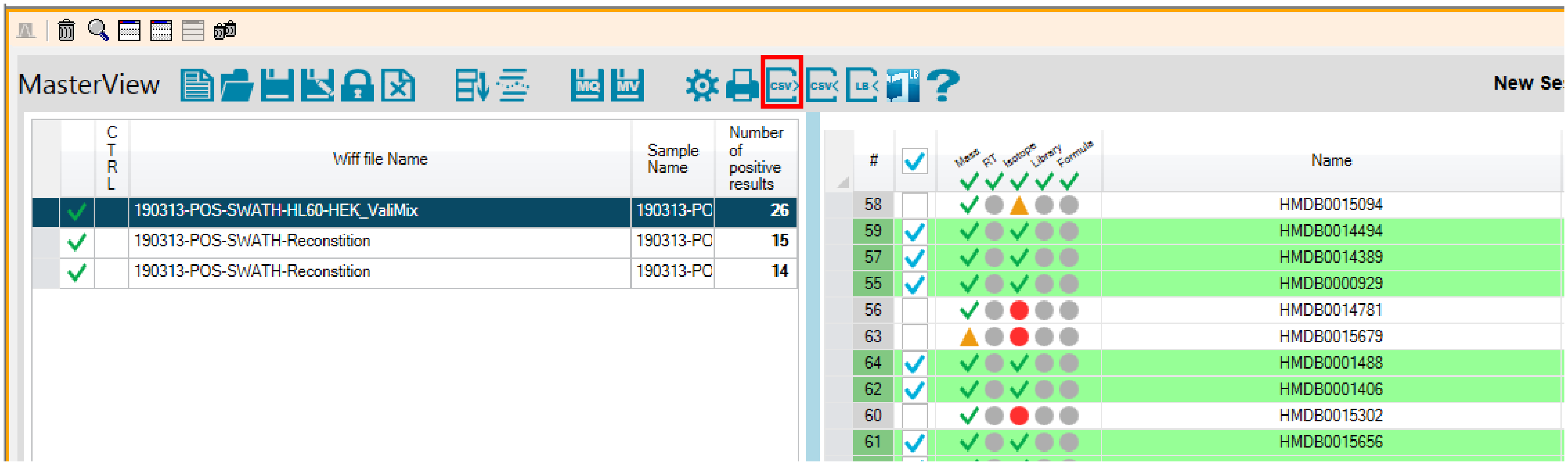

Fig. 16. Result exportation window

8. MultiQuant

1- Open MultiQuant.

2- From File tab >>> Import >>> Quantitation Method from Text…

3- Import the text method generated by SASA from step 3.1.

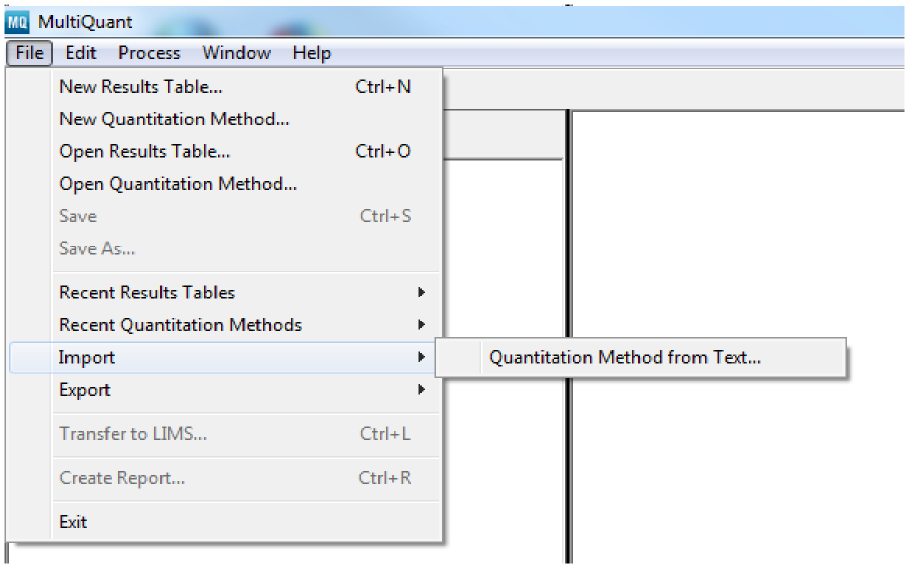

Fig. 17. MultiQuant database uploading

4- Wait until the method opened.

5- From File tab >>> Save as and save the method.

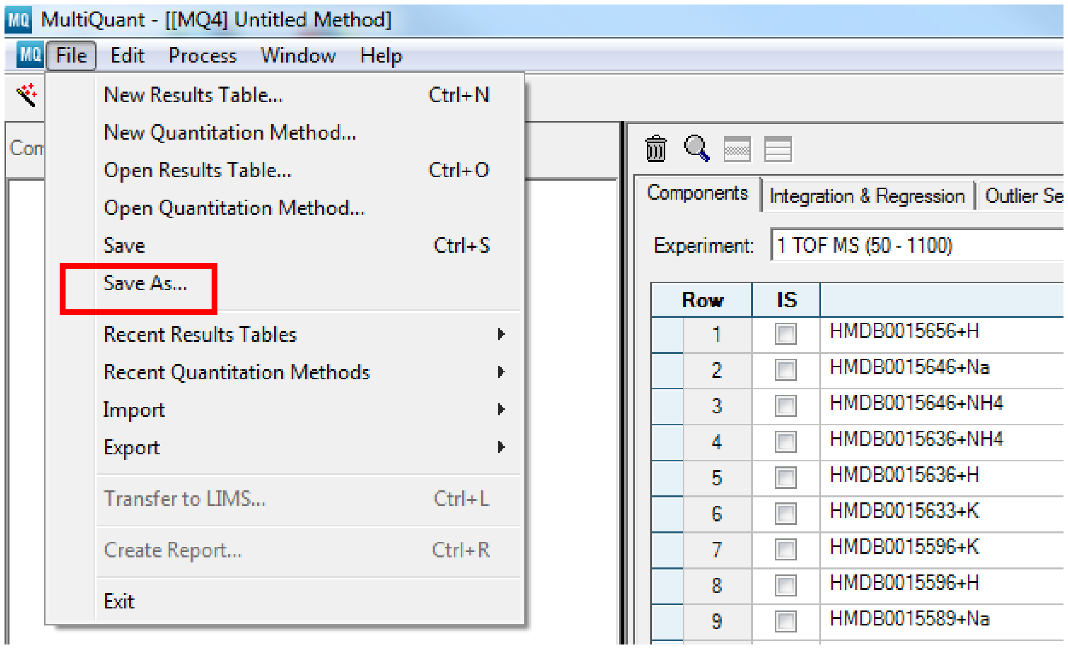

Fig. 18. MultiQuant database generation

6- From the File tab >>> New Results Table >>> Import your sample and the blank >>> Next >>> Choose Existing Method >>> import the method you just saved >>> Finish.

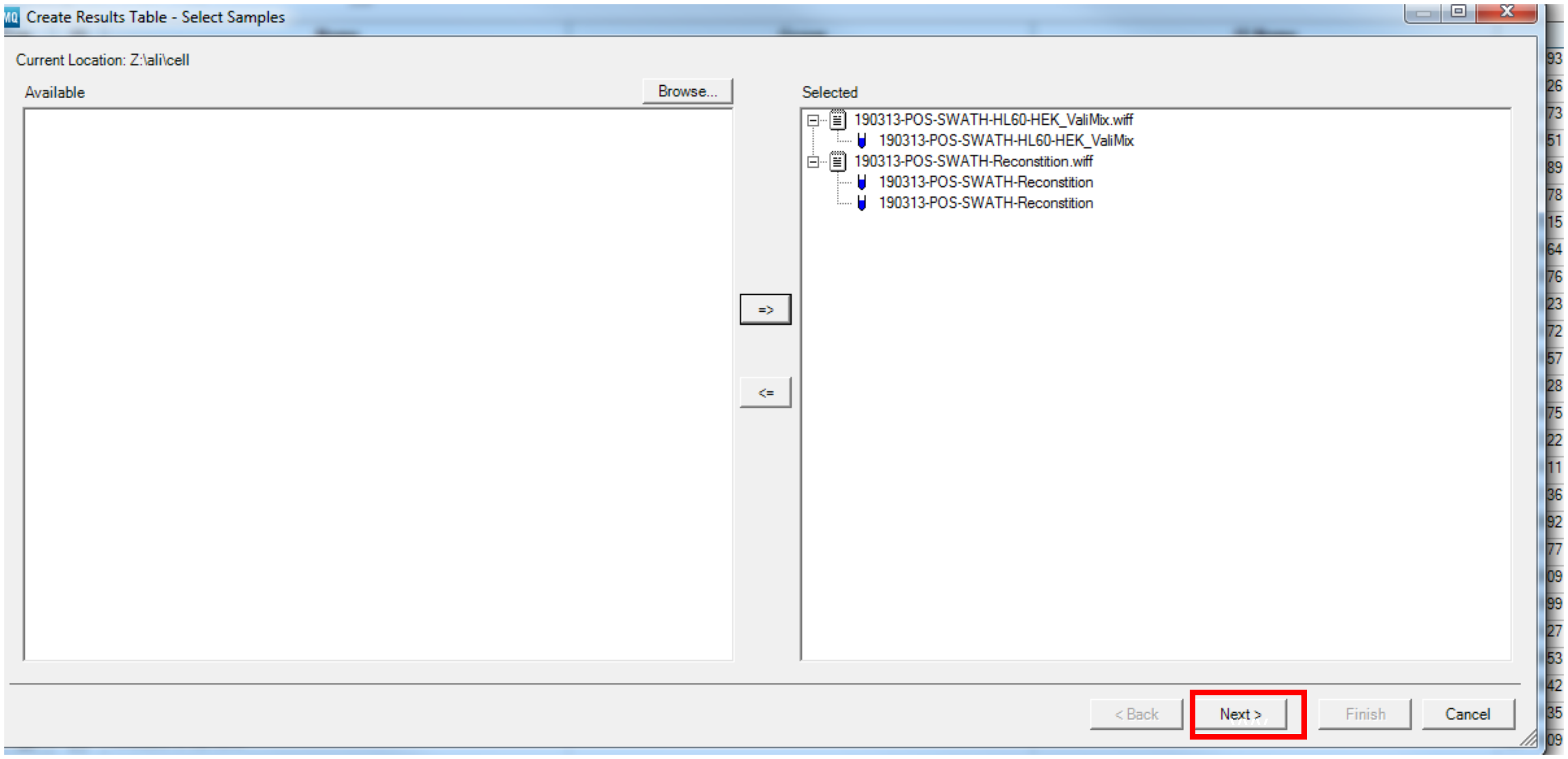

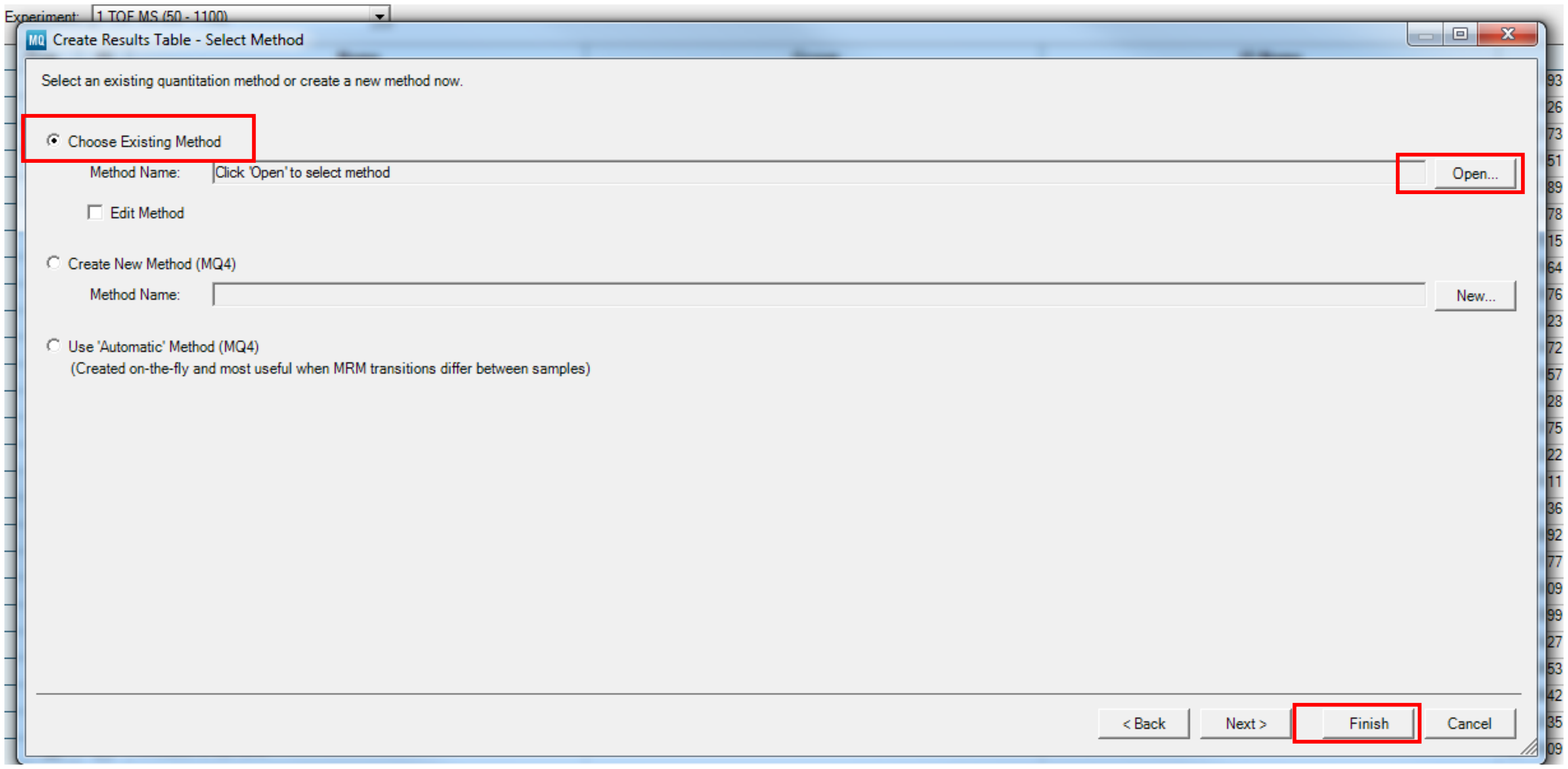

Fig. 19. Uploading of the samples and generated database to MultiQuant

7- Wait until the process is done, this step may take time depend on the size of your sample.

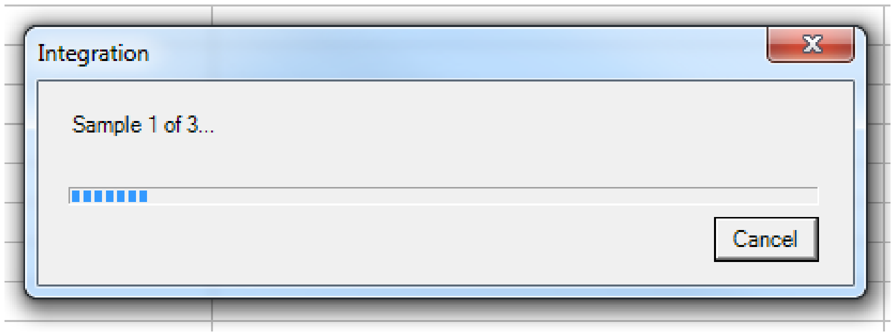

8- From File tab >>> Export >>> Results Table – Metric… >>> select Transpose (sample as columns).

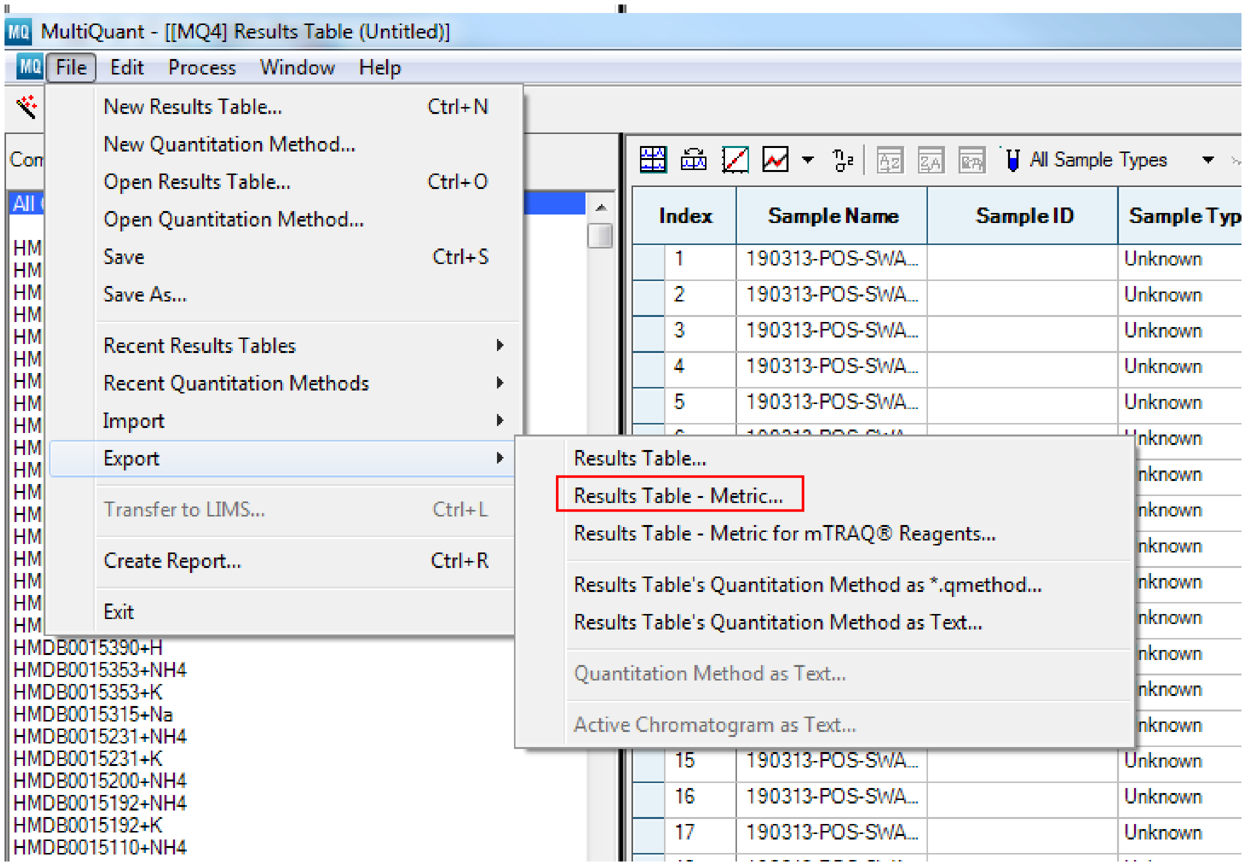

Fig. 20. Exporting the results after retrieving the compound with the generated database

9- Select Transpose window >>> then choose from the Metric Height and save it, then retention time and save it, and finally width at 50% and save it.

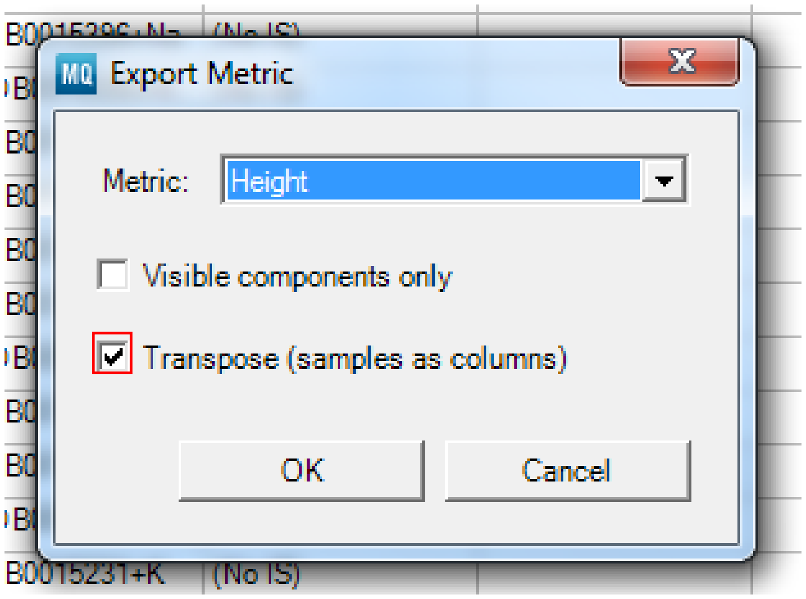

Fig. 21. Exporting metric window

10- By end of this step, in the result output folder will be RT, height, and width 50% will be exist

Built-in Database

There are four built-in database in SASA software:

1- HMDB high resolution positive mode, Experimental. (HMDB HR +ve)
2- HMDB low resolution positive mode, Experimental. (HMDB LR +vs)
3- HMDB high resolution negative mode, Experimental. (HMDB HR –ve)
4- HMDB low resolution negative mode, Experimental. (HMDB LR –ve)

Adducts

In SASA tool we implemented 2 files for adducts; one for positive mode and another for negative mode.

The user can add any other adducts in these files using chemical formula:

- For positive adducts: [M+H]+, change the +H with any other adducts (i.e. [M+Na]+)
- For negative adducts: [M-H]- , change the -H with any other adducts (i.e. [M+Na-2H]-)

	A	B
1	name	massdiff
2	[M+H]+	1.007276
3	[M+Na]+	22.98922
4	[M+K]+	38.96316

Fig. 21. Excel of the different adducts

Demo Files

A folder named “Deme Files” contains all files needed to run a whole process in SASA tool.

1- Database
2- “2. MVmethod.csv” output from step number 2.1.
3- “3. MVmethod.csv_result” output from step number 2.2.
4- “4. MQmethod.txt” output from step number 3.1.
5- “5. MQ.qmethod” output from step number 3.2.
6- “7. Hight” output from step number 3.2.
7- “8. retention time” output from step number 3.2.
8- “9. width at 50%” output from step number 3.2.
9- “10. Filtration_method” output from step number 3.3.
10-“11. Final Result” output from step number 4 (Filtration).

Errors

SASA tried to handle every error that could happen, however some new errors may arise. To handle such case a file named "Errors reported by a compiler" in which any errors and/or bugs reported on it (this file is created in the SASA folder where you installed it). Then one can contact us and send this file to figure out the error and we will try to fix it.

SOFA

SOFA is a user-friendly statistics, analysis, and reporting program. It is free, with an emphasis on ease of use and beautiful output. SOFA lets you display results in an attractive format ready to share. SOFA will help you learn as you go.

From the tool bar you can easily open SOFA and used it, no need to download it.
For more information about SOFA please check: https://www.sofastatistics.com/home.php

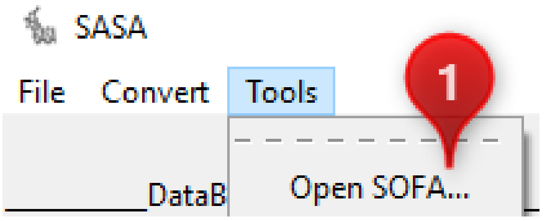

KEEG query

One of the additional tool that SASA provide is KEEG query, which retrieve over KEEG compounds database and compare it with your results and return number of hits.

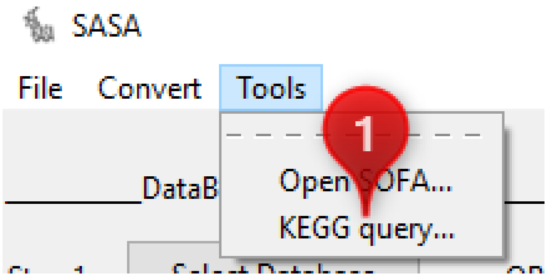

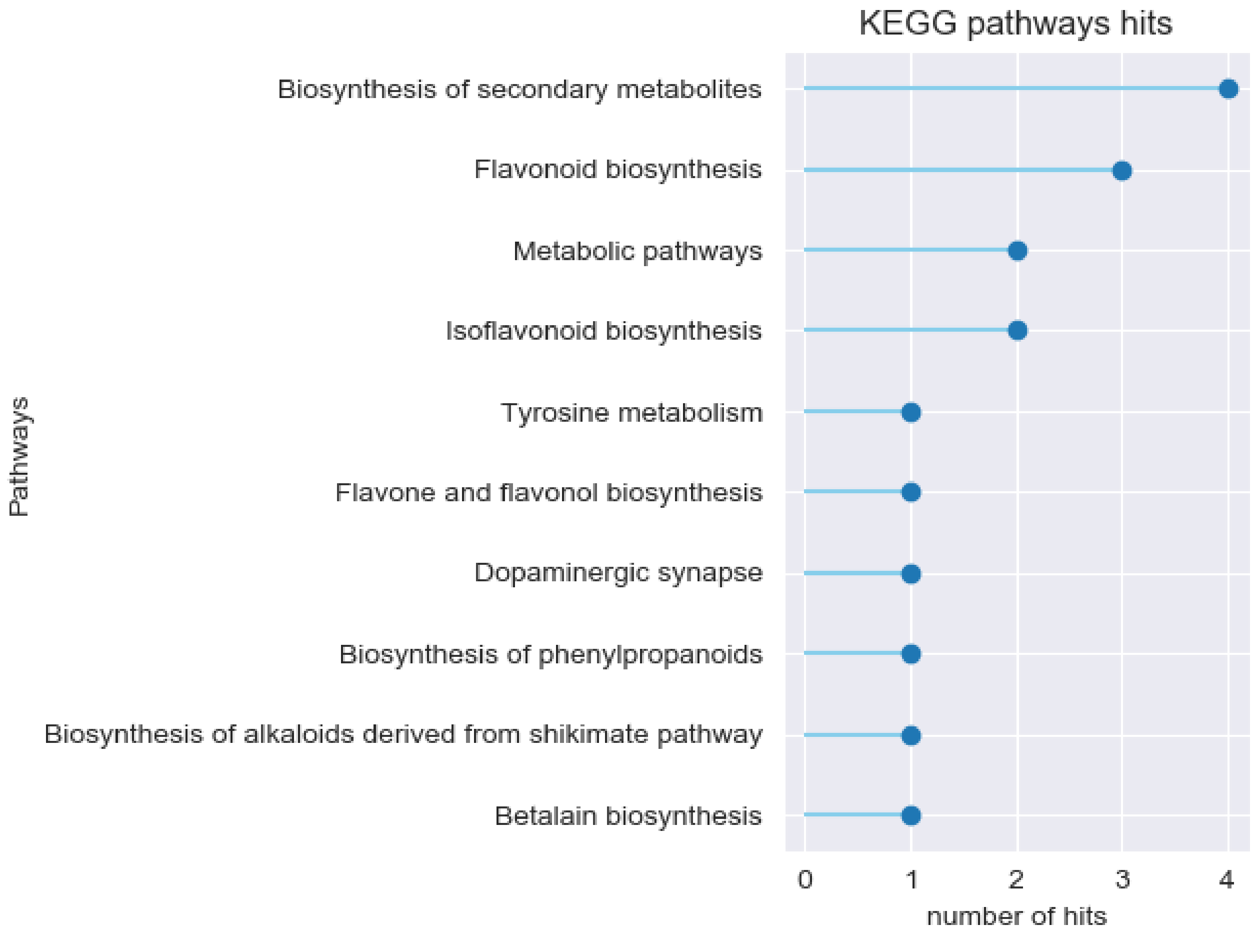

Citation

Note Yet

Copyright (C) 2019-2020 Children's Cancer Hospital 57357 <{proteomics.lab@57357.org}>
